# Prior beliefs & automated fact checking: Limits on the effectiveness of AI-based corrections

**DOI:** 10.1371/journal.pone.0342332

**Published:** 2026-02-05

**Authors:** Kelly M. Amaddio, Jacob T. Goebel, Jason K. Clark, Duane T. Wegener, R. Kelly Garrett, Mark W. Susmann, Srinivasan Parthasarathy

**Affiliations:** 1 Department of Psychology, The Ohio State University, Columbus, Ohio, United States of America; 2 Department of Psychology and Philosophy, Sam Houston State University, Huntsville, Texas, United States of America; 3 School of Communications, The Ohio State University, Columbus, Ohio, United States of America; 4 Department of Psychology, Vanderbilt University, Nashville, Tennessee, United States of America; 5 Department of Computer Science and Engineering, The Ohio State University, Columbus, Ohio, United States of America; Yeditepe University, TÜRKIYE

## Abstract

Proliferation of misinformation poses significant challenges in contemporary society, necessitating efficient strategies for its identification and mitigation. Automated fact-checking systems might prove effective, but they face challenges, particularly in charged contexts where prior beliefs are likely to influence responses to fact-checks. Data from two studies where participants were given a piece of gun-control misinformation and an automated fact-checker correction (*N* = 1,372) illustrate the nuanced interplay between prior beliefs, trust in artificial intelligence (AI), and the perceived accuracy of fact-checking systems in shaping (a) post-correction misinformation endorsement, and (b) post-correction perceptions of system quality. Study 1 examined default perceptions of system accuracy and demonstrated a high degree of variability in those perceptions; when fact-checked by such a system, people’s prior beliefs predicted continued belief after the correction and post-correction perceptions of the fact-check system. Study 2 directly manipulated the purported accuracy of the system. When automated fact-checkers were said to have an accuracy level close to current expectations of existing AI systems (67%), people continued to believe misinformation more to the extent it was consistent with prior beliefs. This pattern was attenuated when participants were told that the fact-checker was highly (97%) accurate. Similarly, prior beliefs related more strongly to post-correction perceptions of system reliability when accuracy information was provided and especially when the system was described as not highly accurate. This research demonstrates biases in reactions to automated fact-checkers and highlights the importance of accounting for individual beliefs and perceived system characteristics in designing scalable interventions.

## Introduction

The Internet, especially social media, allows misinformation to be shared with unprecedented speed and reach, and misperceptions that result can have grave consequences. Because the internet enables (mis)information to be generated and distributed with ease, it is important to identify methods to quickly diagnose misinformation and attempt to limit its impact. The effectiveness of fact checking has been mixed, with some studies finding a reduction in misinformation belief [1,2], others null findings [3], and in some cases even increased belief after fact-checking [4], though subsequent research suggests that this is very rare [5,6]. Meta-analysis has identified moderate influences of corrective messages on reducing belief of misinformation (*r* = .35; [7]), though corrections of political and marketing misinformation were less successful.

Currently, identification of misinformation often involves recruiting fact-checkers to manually evaluate each claim, but this is incredibly time consuming. To thoroughly assess the veracity of a claim, fact-checkers must cross-reference many sources to locate relevant information, evaluate the credibility of those sources, and integrate this information to form a judgment. The process can take professional fact-checkers several hours or days [8,9], making it nearly impossible to keep up with the rate at which (mis)information can be generated. To address this challenge, researchers have been developing automated fact-checking systems [10] using AI technology [11].

Timing matters when it comes to correcting misinformation. When testing the effectiveness of correction placement on Instagram, fact-focused corrections have been more effective when presented after rather than before exposure to the misinformation [12]. However, if corrections after misinformation exposure are delayed, individuals have more time to elaborate and draw causal inferences, which can strengthen belief in the initial misinformation [13] (see also [14–15]). In other words, this additional processing time allows for deeper encoding of the misinformation. Discounting false information works better when the information is less integrated with beliefs and other information stored in memory. Otherwise, judgments continue to be influenced in ways consistent with the discarded information [16]. False beliefs formed from exposure to misinformation require knowledge revision or memory updating, which is cognitively challenging [17]. On platforms like Reddit and Facebook, posts labeled as false by a fact-check were discussed less and for a shorter duration than posts labeled as true [18,19]. Yet, if fact-checks conducted closely after the presentation of the misinformation have the highest likelihood of being effective, it would be important to deliver fact-checking quickly and at scale.

Even if researchers could develop an automated fact checking system that provides judgments of claim accuracy quickly and at scale, it is still possible that individuals would reject those judgments. Perhaps the system’s output conflicts with the recipient’s prior knowledge or beliefs on the topic. Perhaps there are elements of the system that call into question its credibility. Additionally, an individual’s prior experience with AI and automation could affect their level of trust in these types of systems more generally. The research reported here examines these possibilities.

### Consistency of misinformation with existing beliefs

To understand how individuals respond to automated fact checking systems, we start with a brief review of the ways in which they respond to fact checking messages more generally. For example, the effects of political fact check exposure are attenuated by individual variation in preexisting beliefs, political ideology, and domain knowledge [20]. People do not consume information in a vacuum. Rather, they approach new information through the lens of their previous knowledge, motivations, and understanding about how the world works.

Early (mostly cognitive) explanations for continued (post-correction) influence of misinformation included use of misinformation to fill gaps in mental models of the causal understanding of an event [13,21]. Another key determinant of misinformation endorsement likely involves assessment of the fit between the misinformation and other relevant knowledge in memory. Misinformation that is consistent rather than inconsistent with prior beliefs should fit within one’s existing knowledge of the world better, such that corrections of belief-consistent misinformation might be more unexpected and, therefore, surprising or even uncomfortable if it calls into question one’s understanding [1,22–24]. Thus, cognitive and motivational mechanisms are likely to be intertwined. Indeed, motivational approaches suggest that all thinking is motivated [25–28]. That is, “hot” beliefs that are often automatically activated or affective guide subsequent information processing to confirm belief-consistent information and disconfirm belief-inconsistent information [26–29]. Thus, it is perhaps unsurprising that people often continue to believe misinformation after it is corrected, especially when the misinformation is consistent with preexisting attitudes and beliefs (e.g., [4,30,31]). Broader attitudes supporting the misinformation can also provide coherence in the nomological network of beliefs, whereas identification of the misinformation as false can create an uncomfortable gap in understanding [31–32] (cf. [13,21]). Though much of the research alluding to motivated processing does not include measures of discomfort that provide the strongest evidence for motivated processing (cf. [26]), existing studies using such measures suggest that the discomfort can motivate continued belief in the misinformation [31–32].

Misinformation involving politicized topics (like the focal topic in our research -- gun control) can lend itself to motivational reasoning processes generally, but data also show that partisans might fact-check differently. Republicans report more negative perceptions of mainstream media than Democrats [33], and Republican presidential candidates in 2012 and 2016 elections criticized fact checkers as biased [34]. Perhaps relatedly, politically conservative individuals have also been less likely to share politically inconsistent information [35] and more likely to consume and share falsehoods that align with their political beliefs [36–37]. We expect, however, that the motivational and cognitive mechanisms underlying consumption and spread would operate similarly across the political spectrum.

### Correction credibility

It remains unclear how credible automated systems will be perceived by the individuals who encounter them and to what extent specific information related to system credibility might influence the ultimate effectiveness of the corrections. In most situations, automated systems work behind the scenes—functioning as a “black box”—and, as a result, how individuals respond to them depends on their idiosyncratic perceptions of the system’s credibility. We expect wide variability in default perceptions and expect that providing more specific information about system credibility will yield more consistent user perceptions, with implications for the efficacy of these systems in correcting misinformation.

Like human fact checkers, automated fact-checkers are subject to users’ judgments of credibility [38]. When people want to continue believing misinformation, it might be especially important for attempts at correction to come from credible sources if the corrections are to have an impact. Traditionally, the persuasion literature treated credibility as stemming from a combination of source expertise and trustworthiness [39–40], but recent psychological research has identified source bias as an additional determinant of perceived source credibility [41]. Whereas trustworthiness concerns the potential for a source to *intentionally* deceive, judgments about source bias involve considering whether the source has a skewed perspective – that is, a perspective that veers in one direction or another when compared to available information about the topic [42]. Credible sources are typically more persuasive than non-credible sources [43–46], and misinformation corrections are generally more impactful when coming from a credible source (e.g., [47–49]; but see [7]). Liu and colleagues [38] found that regardless of the fact checking source (individuals, news outlets, crowdsourcing, AI), sources perceived as more credible were most effective, and fact-checker credibility moderated the relation between political alignment and fact-checker effectiveness.

### Perceptions of system accuracy

In the automated fact-checking domain, perceived credibility of the system issuing corrections is critically important. In a nationally representative survey of American adults, participants rated fact-checking labels created by algorithms as less effective than ones created by professional fact checkers [50]. Yet, people might weigh various factors differently in their evaluations of automated systems versus human fact-checkers. For instance, the relative accuracy of a system might be more salient than the amount of data that went into the development of the system, whereas amount of knowledge or expertise is a key determinant of credibility for human sources [40,49]. Though automated fact-checking is evolving rapidly, there remains significant room for improvement. A recent review estimated that even the best systems—those trained using a substantial body of domain-specific fact-checking data—were only 65–75% accurate [11]. Though some skepticism of automated fact-checking systems is understandable, efforts to improve fact-checking systems continue [11].

### Trust in automation

Most Americans are unfamiliar with the performance of automated fact checking systems. For these individuals, their default assessments are likely to be informed by their trust in automation more generally: the extent to which an automated agent is anticipated to serve the user’s goals in the face of uncertainty [51]. When an automated system serves the user’s goals, they are likely to view the system as beneficial, useful, and trustworthy, and we directly measured those perceptions in the current research. Such perceptions would parallel key aspects of performance, process, and purpose outlined as bases of trust in automation [51,52]. That is, performance is thought to capture the competency or expertise of the automation in serving the operator’s goals, process reflects the degree to which the automation is appropriate to the operator’s goals, and purpose refers to the automation being used in line with the operator’s intent [53]. To our minds, reports of how beneficial and useful automated systems are tap into participant perceptions of the extent to which the systems provide adequate performance, process, and purpose, with overall perceptions of trustworthiness also capturing a summary perception of how automated systems serve the person’s goals. Because goals in the fact-checking context also include provision of information, these perceptions of utility/capability and trustworthiness should also correspond well to notions of source credibility -- including expertise, trustworthiness, and lack of bias [41–44].

Unsurprisingly, individuals vary in the extent to which they trust machines [54], and trust in automated systems has been identified as a key factor influencing decision-making processes in mixed AI-human groups [55]. Therefore, in our research, we explored the relation between overall trust in automation and default (uninformed) perceptions of the accuracy of an automated fact-checking system. We also examined whether system perceptions, as well as responses to corrections attributed to the system, are influenced more by recipients’ stances toward corrected information depending on their trust in artificial intelligence.

### Biases based in prior stances

Though individuals’ biases shape information processing, obscurity surrounding the details of automated systems might leave even more room for impact of preexisting biases, such as attitudinal [31] or ideological biases [56]. Past research has tended to examine either attitudes on a focal topic or more general political ideology. In the current case, both attitudes and ideology align in particular ways to set up a common stance toward the issue (i.e., with people expressing more liberal ideology and more favorable attitudes toward restrictions on gun ownership representing more favorable stances toward gun control). Though ideology and attitudes can certainly diverge, they tend to align in the current data. That is, as presented in the supplemental materials, ideology and gun control attitudes generally show parallel patterns when analyzed separately, but for efficiency of presentation in the main manuscript, we combined ideology and attitudes into a single *stance* measure.

Biases associated with prior stances on the topic might guide both post-correction misinformation endorsement and post-correction perceptions of the fact-checking system. For example, prior stances could be used as a heuristic to judge the quality of a system that provides feedback consistent (vs. inconsistent) with prior views [57] or could bias the processing of the information available about the automated fact-checking system [58]. The automated nature of the fact-checking system conveys some level of independence and objectivity [59–61], but we suspected that people would be sufficiently skeptical of “black box” automated systems to let those biases hold sway. Another goal of the present research was to examine how people react to fact-checking systems about which they learn more specific information about the quality of the system. In particular, we examined whether information that clearly indicates high system accuracy could successfully weaken the types of bias likely to be evident in the context of default (uninformed) system qualities.

In sum, the primary contributions of this paper are to measure lay perceptions of accuracy of automated fact checking systems, assess whether perceptions of system qualities influence corrective effects of messages attributed to these systems (using both cross-sectional and experimental data), and assess whether prior beliefs bias reactions to fact checking messages and subsequent perceptions of the automated fact checking system.

### Research overview

We conducted a pair of studies. Study 1 examined how accurate people expect an automated fact checker to be when not receiving any specific information about system accuracy (i.e., default perceptions). We expected that these default perceptions would reflect substantial variability and (reasonable) skepticism about the quality of automated fact-checking systems. In other words, we did not expect people to consistently assume that automated fact-checkers are entirely accurate. These relatively uninformed judgements about automated systems should instead be flexible, which set the stage for biases in post-correction misinformation endorsement or in post-correction perceptions of the system. Accordingly, in Study 1 we evaluated whether prior beliefs (such as gun control attitudes and political ideology) affect misinformation endorsement following a correction given by an automated fact checker and, if so, how these beliefs influence post-correction perceptions of the system’s qualities.

Study 2 directly manipulated the purported accuracy of the automated fact checking system. One condition provided information about system accuracy that fell within the range of state-of-the-art automated systems when the research was being conceptualized, whereas the other condition provided participants with information that the system was highly (near perfectly) accurate. We expected that fact checks by a purportedly highly accurate system would be more effective than fact checks by a less accurate system. More importantly, however, we expected that fact checks attributed to the highly accurate system would leave less room for biases from prior beliefs in post-correction misinformation endorsement or post-correction perceptions of system quality.

### Study 1

The aims of Study 1 were to establish baseline expectations for the accuracy of automated fact-checkers, to explore any relations of such perceptions with prior beliefs (i.e., relevant attitudes or political ideology) or with trust in artificial intelligence, and to examine potential biases in post-correction misinformation endorsement or perceptions of the automated system.

## Materials and methods

Both studies were approved by The Ohio State University Institutional Review board and written informed consent was attained from all participants prior to the study. Both studies were performed on the Qualtrics platform, with data analysis conducted using IBM SPSS Statistics, 29^th^ Edition.

### Participants and design

Three hundred and ten adults were recruited on November 1, 2023 from the CloudResearch Connect platform in exchange for monetary compensation. Eight participants were excluded for failing to complete the full study, resulting in a final sample of 302 (*M*_age_ = 37.22; 55.0% male, 42.4% female, 0.3% nonbinary, 2.3% other). Study 1 exposed all participants to the same correction of previously encountered misinformation.

### Procedure

After providing informed consent, participants reported their attitudes toward eighteen objects on 7-point semantic differential scales. Embedded within this battery were three items that together formed an index of gun control attitudes. Trust in artificial intelligence was next measured using another three semantic differential items.

All participants then encountered a stream of information developed for the study that supposedly pertained to the state of Kentucky, including the focal (misinformation) claim about gun ownership and violent crime. The false message asserted that, over a particular period, a reduction in gun ownership led to the lowest rate of violent crime Kentucky has seen in 50 years, a claim that is consistent with support for gun control and inconsistent with opposition to it. We chose to use the topic of gun control because we knew that our U.S.-based sample would find the issue to be important to them and have relatively strong attitudes about the topic. Because of this, we expected their prior beliefs regarding gun control to be strong enough to create relations with post-correction misinformation endorsement [62] (leaving room to potentially reduce relations between prior stances and post-correction endorsement with a correction from a higher quality source). Gun control was also chosen as the focal topic given the substantial variance in gun control attitudes among U.S. participants observed in prior research employing this misinformation paradigm [31,63]. After reading a few filler statements, participants were told that an automated fact-checker would soon analyze the claim about guns and crime. Participants then reported their estimation of how accurate such a system would be. They next saw a correction attributed to the AI fact checker stating that the rate of gun ownership had not actually changed during the period in question, so the reduction in crime was unrelated to gun ownership. All participants then read that the fact-checking system was funded by a non-partisan group (to minimize the potential for recipients to assume a vested interest on the part of system developers when receiving counter-attitudinal corrections). After a couple of additional pieces of information, misinformation endorsement was assessed via a three-item index. Then, twelve semantic differential items measured post-correction perceptions of the qualities of the AI fact-checker, especially regarding potential bias and perceived reliability. Lastly, participants completed several demographic items before being debriefed.

### Measures

#### Gun control attitudes.

Participants indicated how much they favored more restrictive laws on gun ownership (1 = *Strongly Disagree* to 7 = *Strongly Agree*), how good they thought tighter restrictions on gun ownership would be (1 = *Bad* to 7 = *Good*), and how much they agreed more restrictive gun ownership laws would be beneficial (1 = *Strongly Disagree* to 7 = *Strongly Agree*). The same attitude items were used in both Study 1 and Study 2. In each study, responses to the three items were highly correlated. In exploratory factor analyses (EFAs), the three items loaded strongly on a single factor (Study 1 loadings:.95,.96, &.97, respectively; Study 2:.97,.95, &.97), and the Factor Determinacy Index (FDI) [64] was high (Study 1 FDI = .98; Study 2 FDI = .98) -- well above the level identified as adequate for research purposes (i.e.,.8) [65]. Responses to the three items were averaged to form a composite index of gun control attitudes (Study 1: α = .97, *M* = 5.30, *SD* = 2.02; Study 2: α = .97, *M* = 5.11, *SD* = 2.08), with higher values denoting stronger support for gun control laws (see supplemental information for wording of all questions).

### Trust in artificial intelligence

Participants indicated how much they agreed that AI is beneficial to society (1 = *Strongly Disagree* to 7 = *Strongly Agree*), how useful they thought the use of AI in fact checking to be (1 = *Useless* to 7 = *Useful*), and how much they agreed that AI is trustworthy (1 = *Strongly Disagree* to 7 = *Strongly Agree*). The same AI trust items were used in both Study 1 and Study 2. In each study, responses to the three items were highly correlated. EFAs showed that the three items loaded strongly on a single factor (Study 1:.83,.79, &.92, respectively; Study 2:.83,.84, &.89), and the FDI was high (Study 1 FDI = .94; Study 2 FDI = .94). Responses to the three items were averaged to form a composite index of trust in artificial intelligence (Study 1: α = .88, *M* = 4.50, *SD* = 1.49; Study 2: α = .89, *M* = 4.60, *SD* = 1.35).

### Automated fact checker accuracy estimation

Participants were asked to indicate how accurate they thought an AI fact checker would be in analyzing public safety data such as the previously encountered claim. Estimates were reported as a discreet percentage (0% − 100%) on an open-ended response item as well as on a 101-point slider scale. Responses to these two items were averaged to form a composite accuracy estimate (α = .96).

### Post-correction misinformation endorsement

Participants indicated how much they agreed that a decrease in gun ownership led to the reduction in crime in Kentucky (−3 = *Strongly Disagree* to 3 = *Strongly Agree*), how accurate they found that claim to be (−3 = *Extremely Inaccurate* to 3 = *Extremely Accurate*), and how true they thought the misinformation was (−3 = *Extremely Untrue* to 3 = *Extremely True*). The same endorsement items were used in both Study 1 and Study 2. In each study, responses to the three items were highly correlated. EFAs showed that the three items loaded strongly on a single factor (Study 1:.92,.96, &.97, respectively; Study 2:.95,.96, &.95), and the FDI was high (Study 1 FDI = .98; Study 2 FDI = .98). Responses to the three items were averaged to form a composite index of misinformation endorsement (Study 1: α = .96, *M* = −.78, *SD* = 1.83; Study 2: α = .97, *M* = −.53, *SD* = 1.89).

### Post-correction perceptions of fact-checker

Twelve semantic differential items measured how biased and reliable participants thought the AI fact-checker was. Participants indicated the extent to which they thought the automated fact checker: had a biased perspective on gun ownership data (crime statistics) (1 = E*xtremely Unbiased* to 7 = *Extremely Biased*), evaluated gun ownership data (crime statistics) in a biased way (1 = *Not at all* to 7 = *Extremely;* reverse scored), and was biased in evaluating gun ownership data (crime statistics) (1 = *Extremely Biased* to 7 = *Extremely Unbiased;* reverse scored). Then participants were asked the extent to which the automated fact checker’s evaluation of gun ownership data is unduly influenced by its designer’s beliefs or other relevant qualities (1 = *Total Disagreement* to 7 = *Total Agreement*) and whether there is something biased about the automated fact checker that is influencing its evaluation of crime statistics (1 = *Total Disagreement* to 7 = *Total Agreemen*t). The same system bias items were used in both Study 1 and Study 2. In each study, responses to the eight items were reasonably correlated. An EFA showed that the items loaded on a single factor, though the two reverse-scored items did not load as strongly as the other items (Study 1:.87,.88,.90,.91,.48,.52,.84, &.86, respectively; Study 2:.90,.90,.93,.93,.47,.52,.86, &.83, respectively), and the FDI was high (Study 1 FDI = .96; Study 2 FDI = .97). Responses to the eight items were averaged to form a composite index of perceived system bias (Study 1: α = .93, *M* = 3.49, *SD* = 1.33; Study 2: α = .94, *M* = 3.31, *SD* = 1.48).

To measure perceived reliability of the system, four additional questions measured the degree to which participants found the automated fact checker to: provide reliable information (1 = *Extremely Unreliable* to 7 = *Extremely Reliable*), be “knowledgeable” about gun ownership data (1 = *Extremely Ignorant* to 7 = *Extremely Knowledgeable*), be well-informed about gun ownership data (1 = *Extremely Uniformed* to 7 = *Extremely Well-informed*), and to be qualified to evaluate gun ownership data (1 = *Extremely Unqualified* to 7 = *Extremely Qualified*). The same system reliability items were used in both Study 1 and Study 2. In each study, responses to the four items were highly correlated. EFAs showed that the items loaded strongly on a single factor (Study 1:.89,.91,.96, &.91, respectively; Study 2:.87,.92,.94, &.89), and the FDI was high (Study 1 FDI = .97; Study 2 FDI = .97). Responses to the four items were averaged to form a composite index of perceived system reliability (Study 1: α = .95, *M* = 3.61, *SD* = 1.41; Study 2: α = .94, *M* = 4.76, *SD* = 1.36).

### Political ideology

After all experimental manipulations and measures were complete, participants were asked where they fell on the political spectrum (1 = *Extremely Liberal* to 7 = *Extremely Conservative*), *M* = 3.68, *SD* = 1.65. To facilitate combination and comparison with gun-control attitudes, this item was reverse-scored such that higher values are associated with more liberal views.

## Results

### Analysis strategy

Political ideology and gun control attitudes were strongly correlated, *r (*301) =.56, *p* < .001. Consistent with polling data, participants who identified as more politically liberal were more in favor of gun control measures [66]. Previous misinformation research has typically examined either attitudes or political ideology as background beliefs with which misinformation might be relatively consistent or inconsistent. One could hold a stance on gun control partly because they believe the stance has merit on its own and partly (overlappingly) because it is consistent with other related stances captured by political ideology. Because the current research was not aimed at distinguishing which of these correlated background beliefs drive post-correction endorsement, initial analyses used a composite “stance” variable created by averaging the attitude composite measure and the political ideology measure (reverse coded) so that higher values indicate more liberal/pro-gun-control views. In this paper, we use the term “stance” to indicate an average of political ideology and gun control attitudes. It is important to note, however, that the term “stance” is used differently in other areas like computer science (e.g., [67]). We primarily created the stance variable as an efficient way to convey the prior beliefs that might form the backdrop for biases in post-correction perceptions of the misinformation or perceptions of the automated fact-checking system. Analyses using only attitudes or only political ideology produced mostly parallel effects to the stance analyses, but analyses that included both factors separately produced few independent effects (suggesting that most of the impact of prior stances represent the shared prior beliefs reflected in both attitudes and political ideology). Analyses using only attitudes or only ideology are reported in the supplemental materials.

Though our primary focus was on how background beliefs influence continued endorsement of the misinformation after fact-checking and perceptions of the automated system, stances on the topic could differ across demographic categories like age, gender, and race. We examined potential relations between participant stances and the demographic variables we measured. Participant stances showed no significant relations with any of the demographic variables, so the following analyses for Study 1 were conducted without controlling for these factors. For each analysis throughout the manuscript, we examined all participants who provided responses to all the measured variables in that analysis. Therefore, differences in degrees of freedom across analyses of different outcome variables reflect differences in how many people completed that outcome measure.

### Automated fact-checking system accuracy estimates

On average, participants’ estimates of system accuracy resembled the current state of automated fact-checking, but with high variance [11]. That is, participants estimated the accuracy of the system at just over 70%, but with considerable variation around that average perception (*M* = 70.42, *SD* = 19.56). We conducted a general linear model (multiple regression) analysis predicting system accuracy estimates using participant stance and trust in AI. Results indicated significant main effects of trust in AI, *b* = 6.12, *t*(299) = 9.24, *p <* .001, *r* = .47, and stance, *b* = 1.72, *t*(299) = 2.82, *p = .*005, *r* = .16. That is, people who trusted AI believed that the system was likely to be more accurate, and people who held more conservative/anti-gun-control stances were more skeptical of system accuracy before receiving any correction.

### Post-correction misinformation endorsement

A general linear model (multiple regression) analysis estimated post-correction endorsement of the original pro-gun control misinformation using trust in AI and participant stances as predictors. Results revealed a significant main effect of participant stance on post-correction misinformation endorsement, *b* = .29, *t*(299) = 4.54, *p* < .001, *r* = .25, but trust in AI did not significantly predict post-correction misinformation endorsement. Thus, following identification of a piece of misinformation as false by an automated fact checker, there was a significant tendency for people to continue to endorse the misinformation to the extent that the misinformation aligned with their stance on the issue.

### Post-correction reliability perceptions of fact-checker

Participants’ perceptions of automated fact checker accuracy were positively correlated with their post-correction reliability perceptions of the fact-checker, *r (*300) =.37, *p* < .001. Those who had higher expectations of accuracy perceived the fact checker as more reliable post-correction. We conducted a general linear model (multiple regression) analysis predicting post-correction reliability perceptions using trust in AI and participant stances as predictor variables. Trust in AI significantly predicted post-correction reliability perceptions, *b* = .47, *t (*299) = 9.82, *p* < .001, *r* = .49, but participant issue stance did not, *b* = −.019, *t (*299) = −.42, *p* = .669, *r* = .02.

### Post-correction bias perceptions of automated fact-checker

We conducted a general linear model (multiple regression) analysis predicting post-correction bias perceptions of the automated fact checker using trust in AI and participant stances as predictor variables. Trust in AI significantly predicted post-correction bias perceptions, *b* = −.25, *t (*299) = −5.02, *p* < .001, *r* = .28, but participant issue stance did not, *b* = −.04, *t (*299) = −.89, *p* = 0.38, *r* = .05.

## Discussion

This study provides several important insights. First, the average level of accuracy that participants expected from the automated fact checker (~70%) closely parallelled a state-of-the-art assessment by experts at the time the research was conducted [11]. The perceived accuracy of the system was low enough that participants could easily use it to justify disregarding the corrections provided. Consistent with this notion, post-correction misinformation endorsement was highly correlated with participants’ prior stances on the issue, which we take as evidence of a bias in assessment of the misinformation. This result parallels previous research focused on fact checking attributed to humans, which associated post-correction misinformation endorsement with attitudes (e.g., [31]) or political ideology (e.g., [68]). Additionally, we found that post-correction perceptions of system bias and reliability were significantly associated with participant trust in AI, but (perhaps surprisingly) not significantly associated with prior stances on the issue.

### Study 2

Despite relatively unbiased post-correction perceptions of the fact-checking system, post-correction endorsement of the misinformation was biased. Was this because no specific information about system quality was provided? Were participants’ default perceptions of system accuracy low enough that biases emerged more readily in their judgments (e.g., being more likely to dismiss the correction when the misinformation fit with one’s prior stance on the issue)? To examine these questions, we provided *a priori* information about system accuracy, varying whether the quality of the system was similar to current state of the art (which seems assailable, as in Study 1) or was actually much higher. To this end, participants in Study 2 were either informed that system accuracy closely matched the current state of the art, which was also close to the average expected accuracy level in Study 1 (~70% accurate), or that the system was instead highly accurate (~100% accurate)—a mild deception. This design allowed us to assess whether “infallibility” attributed to the system can attenuate biases on post-correction misinformation endorsement based on participants’ prior stance on the issue. If so, this would also provide additional evidence that individuals use the perceived fallibility of an automated fact checking system to justify dismissal of attitude-inconsistent corrections. Such a possibility would suggest an interaction between stance and system accuracy, with one’s prior stance playing a bigger role in determining post-correction misinformation endorsement when system accuracy is relatively low rather than high.

In situations involving more specific information about the fact-checking system’s accuracy, it is not clear that participants’ trust in AI more generally would continue to shape the influence of the fact checking messages. It could be that information about the reliability of the automated fact checking system might trump attitudes toward AI; alternatively, it is possible that generalized distrust toward AI might still be used to cast doubt on the corrections, especially when the misinformation fits with one’s prior beliefs. If so, this would imply an interaction between trust in AI and stance in which the stance variable would be more strongly related to post-correction misinformation endorsement when trust in AI is relatively low rather than high.

## Materials and methods

### Participants and design

A total of 1107 adults were recruited from the Amazon’s Mechanical Turk platform from June 12^th^ to August 25th, 2023, in exchange for monetary compensation. Thirty-five participants were excluded for failing to complete the full study, resulting in a final sample of 1072 (*M*_age_ = 42.32, 45.7% male, 52.9% female, 1.4% other). Initial pilot data (N of approximately 250) using a simple characterization of accuracy of 97% versus 67% suggested that effects of AI characteristics were likely to be relatively small, so we attempted to strengthen the manipulation (i.e., by characterizing the 97% accurate system as industry leading and the 67% accurate system as a beta version). We collected a second pilot of approximately 250 subjects and found in manipulation checks (i.e., system reliability ratings) that the change in the manipulation made very little difference in the extent to which the high-accuracy system was viewed as more reliable than the low-accuracy system. Rather than continue to revise the manipulation, we used the second (intended to be stronger) manipulation and collected additional data to reach approximately 1000 participants total (across all data collections) who provided complete responses. More participants began the study than finished, and we ended up with 1072 complete responses overall.

Study 2 participants all received the same misinformation and correction used in Study 1. However, the accuracy of the supposed fact checking system was experimentally manipulated to be relatively high or low.

### Procedure

The procedure paralleled Study 1. However, participants were not asked to provide their own estimate of the accuracy of automated fact checking systems. Instead, following the misinformation correction, participants encountered a manipulation of the system’s purported accuracy. As in Study 1, all participants then learned that the fact-checking system was funded by a non-partisan group. This allowed us to hold constant the vested interest of the correction source.

### Measures and manipulations

#### Predictor measures.

Measures of gun control attitudes, political ideology (*M* = 3.70, *SD* = 1.83), and trust in AI were identical to Study 1.

### Outcome measures

Post-correction measures of misinformation endorsement, post-correction perceptions of system reliability, and system bias were identical to Study 1.

### Accuracy manipulation

Study 2 included two almost identical accuracy manipulations. The initial manipulation simply identified the high-accuracy system as 97% accurate and the low-accuracy system as 67% accurate. The initial pilot data suggested that the effects of the system manipulation might be fairly small, so we attempted to strengthen the manipulation for the rest of the participants. The only difference between the two manipulations was that the second manipulation included a short descriptor of the automated fact checking system as “industry leading” in the high-accuracy (97% accurate) condition or as a “prototype” system in the low-accuracy (67% accuracy) condition. The intent was to strengthen the manipulation of system credibility in the second data collection, but manipulation checks found no statistically significant differences in perceptions of system reliability between the two manipulations. Thus, we analyzed the datasets together (and none of the key patterns differed in substantive ways between the datasets if analyzed with manipulation type as a factor in the regressions).

Participants were randomly assigned to a low accuracy or high accuracy condition. In the low accuracy condition, after being given the assessment by the automated fact checker, participants read that “this (prototype) automated fact checker was determined to be 67% accurate in analyzing public safety data.” In contrast, those assigned to the high accuracy condition read that “this (industry-leading) automated fact checker was determined to be 97% accurate in analyzing public safety data.” As noted earlier, the material in parentheses was added in an attempt to strengthen the manipulation, but the pattern of results was generally consistent across the two types of manipulations.

## Results

### Preliminary data analysis

To be as transparent as possible about the different data collections that we undertook, we analyzed the data taking into account the differences across datasets in two different ways. First, we included the type of system accuracy manipulation (i.e., “97% accurate” vs “97% accurate and industry leading”) as a factor in the primary regression analyses. Second, we conducted an analysis that split the pilot from the additional data with the revised accuracy manipulation to separate the three chronological times at which data collection occurred. Because the type of manipulation seemed to constitute the more substantive distinction, the main text reports the analyses using the type of accuracy manipulation as an additional factor in the analysis. We also placed the analysis splitting the three chronological times of data collection in the supplemental materials. In each case, there was no moderation across manipulation types or chronological time for the key stance x accuracy condition interaction. As described later, the stance x trust in AI pattern was moderated by the type of manipulation, but the interaction was significant in both instantiations (i.e., the moderation showed that the pattern was stronger in one instantiation than the other, though we did not attempt to interpret this pattern, because the effect being moderated by type of manipulation did not include effects of the system accuracy conditions).

Although our primary focus was on how prior participant stances influence misinformation endorsement and source perceptions, we also realized that stances could differ across demographic categories like age, gender, and race. Among these three demographic variables, gender was the only one where differences in stances were found (such that participants who identified as male were more anti-gun-control and conservative than participants who did not identify as male). However, analyses that controlled for gender produced very similar results to those not controlling for gender (with no different conclusions than those not controlling for gender). Therefore, in the following sections, we present analyses not controlling for gender.

### Post-correction misinformation endorsement

We conducted a general linear model (regression) analysis that included the following terms as mean-centered predictors of post-correction misinformation endorsement: accuracy condition, stance, trust in AI, and type of accuracy manipulation. Across analyses, we examined the full model including all interactions. Because the stance, trust in AI, and type of accuracy manipulation terms were not necessarily symmetric (thereby creating relations between the lower and higher-order terms including the predictor variable), we could not run a simultaneous full factorial analysis. Rather, we obtained numerator mean squares in a stepwise fashion from models including only main effects, main effects plus two-way interactions, main effects plus two- and three-way interactions, and then the full four-way model. The denominator mean square for the error term was obtained from the full model analysis. Because all terms had 1 degree of freedom in the numerator, all t values reported in the following sections are the square root of the F values constructed by dividing the numerator mean square by the denominator mean square. A table reporting significance values for all terms in the model appears in the supplement.

There was a significant main effect of accuracy condition such that corrections by a more accurate system resulted in less post-correction endorsement of the misinformation, *b* = −.51, *t*(1055) = −4.85, *p* < .001, *r* = .15. There was also a main effect of stance such that participants who were relatively liberal and supportive of gun control continued to endorse the pro-gun-control misinformation after the correction more than those who were relatively conservative and against gun-control, *b* = .35, *t*(1055) = 11.47, *p* < .001, *r* = .33. There was also an overall difference between the level of post-correction misinformation endorsement following the stripped down (97% accurate) manipulation and the elaborated (97% and industry leading) manipulation. Misinformation endorsement was lower overall with the more elaborated manipulation (which could also reflect the different time points at which data were collected), *b* = −.62, *t*(1055) = −4.91, *p* < .001, *r* = .15.

**Fig 1:**
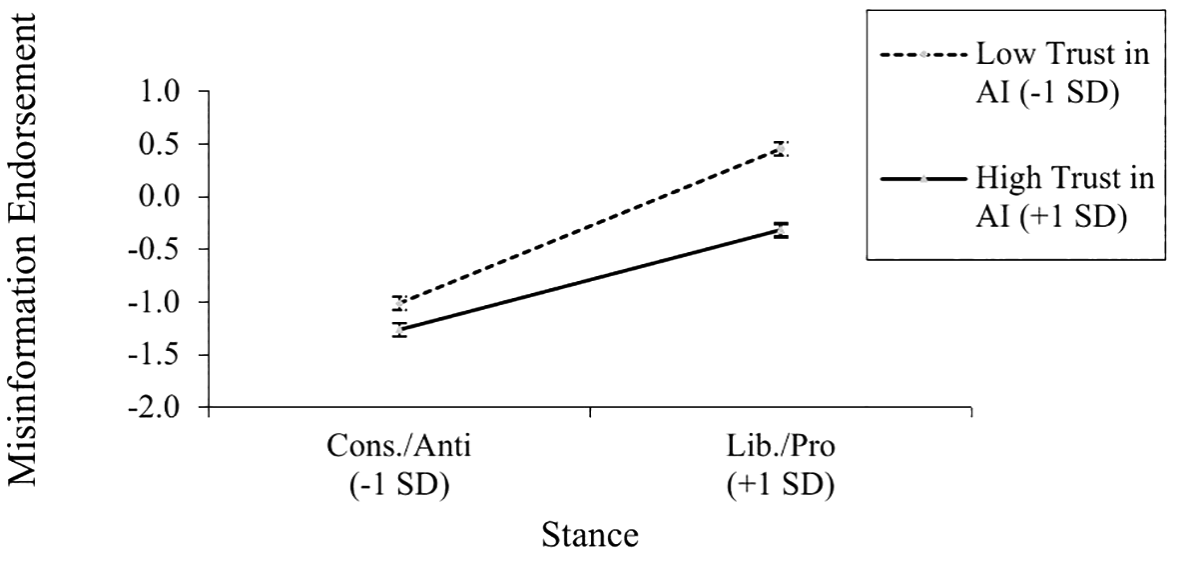
Misinformation Endorsement as a Function of System Accuracy and Participant Stance. Stance was more related to misinformation endorsement when system accuracy was low (67% accurate) rather than high (97% accurate).

More importantly, these main effects were qualified by two key interactions. First, the accuracy condition significantly interacted with participants’ prior stance on the issue, *b* = .15, *t*(1055) = 2.37, *p* = .018, *r* = .07. As shown in Fig 1, participants’ prior stance was more strongly related to post-correction misinformation endorsement when system accuracy was manipulated to be relatively low, *b* = .43, *t*(1055) = 8.44, *p* < .001, *r* = .25, rather than high, *b* = .28, *t*(1055) = 6.14, *p* < .001, *r* = .19. As shown in Tables S2 and S3 in S1 File in the supplement, this pattern was quite consistent for both attitudes and ideology as sources of participant stances.

**Fig 2:**
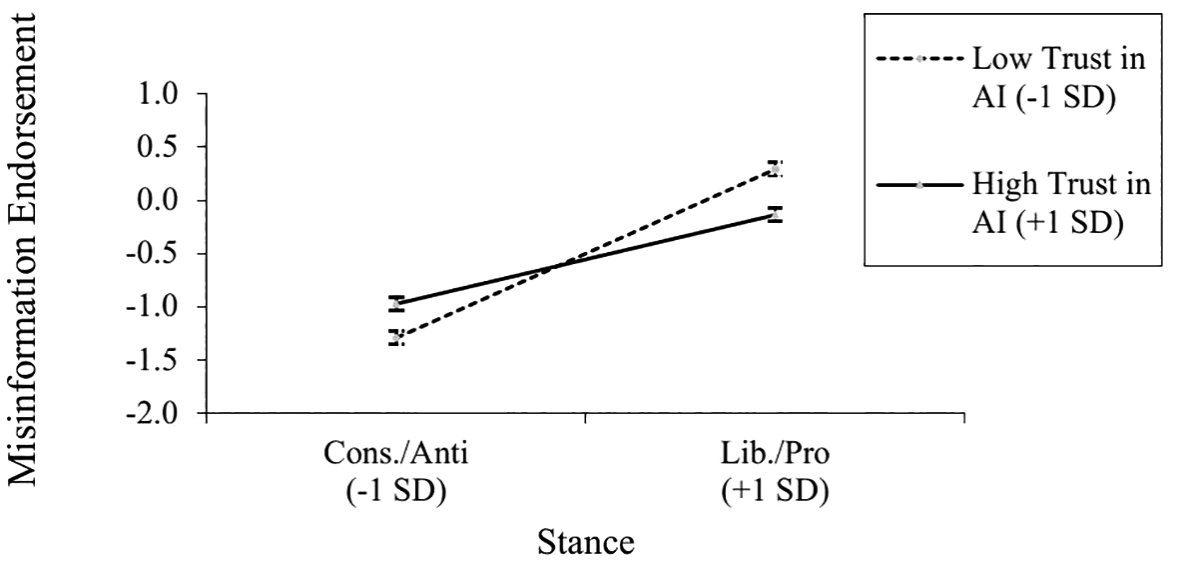
Misinformation Endorsement Based on Participant Stance and Trust in Automation. *Stance predicted endorsement of misinformation more strongly as trust in AI decreased*.

As suggested by the notion of correction source fallibility, there was also a significant interaction between trust in AI and stance, *b* = −.11, *t*(1055) = −3.28, *p* = .001, *r* = .10. As shown in Fig 2, holding a more liberal stance predicted endorsement of the pro-gun control misinformation more strongly when trust in AI was relatively low (−1 *SD*), *b* = .42, *t*(1055) = 8.47, *p* < .001, *r* = .25, than when trust in AI was relatively high (+1 *SD*), *b* = .13, *t*(1055) = 2.62, *p* = .009, *r* = .08. As shown in the supplement, this pattern was quite strong for ideology as a source of participant stances (Table S3 in S1 File) but not for attitudes (Table S2 in S1 File).

The trust in AI x stance interaction varied in strength across the different types of accuracy manipulation such that the interaction was stronger when the elaborated accuracy manipulation was used than when the stripped-down manipulation was used, *b* = .11, *t*(1055) = 2.13, *p* < .033, *r* = .07. The overall relation between trust in AI and post-correction misinformation endorsement was also stronger when the stripped-down accuracy manipulation was used than when the more elaborated system accuracy manipulation was used, *b* = −.51, *t*(1055) = −5.48, *p* < .001, *r* = .17. Because we had no predictions related to type of accuracy manipulation and there was no difference in the effect of the accuracy manipulation on perceived reliability across manipulation types (F < 1), we did not seek to interpret these patterns. No additional effects approached significance *(ps* ≥ .21).

### Post-correction system reliability perceptions

We followed the same stepwise general linear model procedure as in the misinformation endorsement analysis. There was a significant main effect of the accuracy manipulation on post-correction reliability perceptions where those who encountered the high accuracy system rated it as more reliable than those who encountered the low accuracy system, *b* = .40, *t*(1055) = 5.88, *p* < .001, *r* = 18. There was also a main effect of stance such that participants who were more liberal/pro-gun-control viewed the system as less reliable than participants who were more conservative/anti-gun-control, *b* = −.13, *t*(1055) = −6.59, *p* < .001, *r* = .20. Thus, the system was perceived as more reliable when it aligned with what people wanted to hear. There was also a significant main effect of trust in AI where those who were more trusting in AI found the system to be more reliable, *b* = .570, *t*(1055) = 22.17, *p* < .001, *r* = .56.

There was a significant two-way interaction between stance and accuracy condition that paralleled the post-correction endorsement of misinformation, *b* = −.09, *t*(1055) = −2.31, *p* = .021, *r* = .07. That is, stance was more strongly related to post-correction reliability perceptions for the low system accuracy condition *b* = −.18, *t*(1055) = −4.23, *p* < .001, *r* = .13, than for the high system accuracy condition, *b* = −.08, *t*(1055) = −2.86, *p* = .004, *r* = .09. As shown in the supplement, this pattern was similar across attitudes (Table S5 in S1 File) and ideology (Table S6 in S1 File) as sources of participant stances, though the pattern was significant using ideology but not when using attitudes as a predictor. An interaction between accuracy condition and trust in AI also approached but did not reach statistical significance, *b* = −.09, *t*(1055) = −1.80, *p* = .074, *r* = .06. The pattern was such that perceived reliability was more different across accuracy conditions when trust in AI was high rather than low.

### Post-correction Bias Perceptions

We followed the same stepwise general linear model procedure as the misinformation endorsement and reliability perception analyses. The results revealed a significant main effect of the accuracy manipulation such that the more accurate system was viewed as less biased, *b* = −.29, *t (*1054) = −3.60, *p* < .001, *r* = .11. There was also a main effect of trust in AI such that those who were less trusting of AI found the system to be more biased, *b* = −.27, *t*(1054) = −8.58, *p* < .001, *r* = .26. Additionally, a significant main effect of manipulation type was found where the system with the stripped down (97% accurate) manipulation was seen as more biased than the elaborated (97% and industry leading) manipulation, *b* = −.43, *t*(1054) = −4.38, *p* < .001, *r* = .13. Of note, the main effect of stance on bias perceptions approached but did not reach significance, *b* = .04, *t*(1054) = 1.81, *p* < .07.

These main effect patterns were qualified by a significant two-way interaction between accuracy condition and trust in AI, *b* = −.21, *t*(1054) = −3.42, *p* < .001, *r* = .11, such that the effect of the accuracy manipulation was stronger with higher trust in AI. Additionally, there was a significant two-way interaction between stance and trust in AI, *b* = −.043, *t*(1054) = −2.44, *p* = .01, *r* = .08, such that the effect of stance on perceived bias was stronger with lower trust in AI. As shown in the supplement, this pattern was quite weak when using gun control attitudes as a source of participant stances (Table S8 in S1 File) but was quite strong when using participant ideology (Table S9 in S1 File).

Interestingly, there was a significant two-way interaction between trust in AI and manipulation type, *b* = −.29, *t*(1054) = −3.96, *p* < .001, *r* = .12, and a significant three-way interaction between trust in AI, manipulation type, and stance, *b* = .10, *t*(1054) = −2.48, *p* = .01, *r* = .08. The 3-way interaction showed that the trust in AI x stance interaction was stronger with the more elaborated manipulation of system accuracy. However, no other predictors in the model were significant (*ps* ≥ .23). Thus, the key accuracy condition x stance interaction that appeared for misinformation endorsement and perceived system reliability did not appear on perceived system bias.

## Discussion

When given specific information about system accuracy, elevated levels of system accuracy dampened the relation between participant stance and post-correction endorsement of the misinformation. In other words, it seems that participants used negative information about system accuracy to discredit corrections that contradicted their stance on the issue. Thus, explicitly attributing an elevated level of accuracy to the system attenuated attitudinal biases by making it more difficult for these individuals (i.e., especially those for whom the original misinformation was attitude-consistent) to discredit the correction source. Similar to the system being undermined by low accuracy information, prior stance was also more related to post-correction endorsement of the misinformation when trust in AI was relatively low rather than high.

Interestingly, participants who were more liberal/pro-gun-control viewed the system as less reliable following the correction than those who were more conservative/anti-gun-control. This is a departure from previous literature on AI credibility perceptions. However, the pattern is consistent with the literature on confirmation bias and misinformation, suggesting that people’s biased perceptions are more strongly influenced by what they want the system to say rather than their default perceptions of automated systems. This pattern is consistent with the notion of recipients discrediting the source of a correction that conflicts with what they might prefer. Another potential explanation of these findings is that participants are engaging in Bayesian updating. Because they likely have little prior information about the automated system, they put more weight on their knowledge about gun control and use that to inform their assessment of this novel system [69]. The impact of prior beliefs on perceptions of system reliability were also stronger when accuracy of the correcting system was relatively low rather than high. Also, for both perceptions of system reliability and bias, influences of the system accuracy manipulation tended to be stronger when trust in AI was relatively high rather than low.

### General discussion

The current studies showed that participant stance on gun control related to post-correction endorsement of the misinformation, and this relation was stronger when perceived accuracy/reliability of the automated fact-checking system was relatively low (in Studies 1 & 2) than when perceived accuracy/reliability of the system was high (Study 2). Study 2 also showed that stance was related to perceptions of system reliability, consistent with the notion that people discredit a system that provides a correction inconsistent with their worldview. However, this pattern was especially evident when information about the system was somewhat ambiguous (i.e., a 67% accurate system) rather than unambiguous (i.e., 97% accurate). The results of this study have clear implications for the effectiveness of automated fact checkers. Even when automated fact checkers are reported to be 97% accurate, people are still likely to discount them at least to some degree when their conclusions are inconsistent with prior beliefs. That is, even with a high-accuracy fact-checking system, people continue to believe the misinformation more post-correction and also tend to view the system as somewhat less reliable when the correction conflicts with their prior stance on the issue.

Additionally, this research highlighted the fact that people currently exhibit a wide range of expectations about the accuracy of automated fact checkers. Although the mean expected accuracy level was 70% in Study 1, the standard deviation of 19 percentage points and range spanning from 0% all the way to 100% implies considerable variance in people’s expectations. This, combined with the effectiveness of our accuracy manipulation in Study 2, suggests that, at least for now, perceived reliability can be substantially influenced by information about the automated system. Because people often know little about the characteristics of an automated system, there are many ways in which new information could influence perceptions of the system. For instance, future work might profitably examine how information about the data on which a fact checker was trained might influence perceived system reliability.

Previous research in our lab using no-correction control groups and comparing them with human correction sources suggests that prior gun control stances predict control-condition misinformation endorsement more strongly than when a correction is present. That is, across several data collections (approximately 1500 participants) with no correction of the misinformation, the relation between prior gun control attitudes and misinformation endorsement was quite strong (r = .44). In the current Study 2, the relations between the same prior gun control attitudes and misinformation were somewhat weaker when there was a correction from a low-accuracy automated system (r = .34) and especially when the correction came from an automated system high in accuracy (r = .23). Therefore, if we had included a no-correction condition in our current Study 2, we would have expected a relatively stronger relation between gun control stances and misinformation endorsement than in the current low system accuracy condition. However, such a relation would not have been germane to the current research question of whether a low-accuracy versus high-accuracy automated system would create different effects of prior stances on post-correction misinformation endorsement. Therefore, we did not include a no-correction control condition in the current research.

Of course, perfectly accurate automated fact checking systems are far out of reach—even trained human experts make mistakes—yet, automated systems are likely to develop over time (though at different rates for different domains or systems). One risk is that these malleable perceptions might be exploited by actors who benefit from promoting confusion. Because popular views on system accuracy have yet to converge, a malicious actor might attempt to manipulate public perceptions of system accuracy to diminish their future effectiveness. Thus, future research might also focus on how indirect experiences of automated fact-checking might inform the automated fact checker’s credibility. Regardless, care should be taken to discourage overly optimistic or pessimistic perceptions of AI-powered systems. Rather, a critical approach to evaluating these systems is needed, one fostered by efforts to increase AI literacy among the public. Such literacy might attenuate the threat of attacks on the legitimacy of automated fact-checkers by actors with malicious intent.

Previous research has noted how people with a politically conservative orientation can view fact-checking more negatively than people with a more liberal orientation [34]. The results of Study 1 accorded with this pattern—more liberal participants’ default (uninformed) ratings of the automated system depicted the system as likely to be more accurate than ratings by more conservate participants. However, our results also suggest that prior beliefs relevant to the messages being assessed can influence perceptions of fact-checking systems. In Study 2, when the purported system accuracy level was provided, post-correction perceptions of system reliability were guided by how the system’s fact-check fit with prior beliefs about gun control. That is, relatively conservative perceivers (who were also less favorable toward gun control) viewed the system as more reliable than relatively liberal perceivers after the system identified the pro-gun-control misinformation as false. This pattern was strongest when the system was ascribed relatively low accuracy rather than high accuracy, but the pattern was present in each condition. In other words, perceptions of the system were seemingly influenced more by prior beliefs on the issue rather than their default views of automated system accuracy in general.

Future research could certainly do more to address the specific cognitive and motivational mechanisms responsible for the observed patterns. For example, as described in the introduction section, corrections to misinformation can represent cognitive expectancy violations or create gaps in a mental model of causality, and prior beliefs could be used in relatively heuristic ways or could bias more effortful processing of available information about the automated system. Intertwined with these cognitive mechanisms are potential motivations to maintain coherent sets of beliefs and to seek out new information that fosters that sense of coherence. In line with that conceptualization, we do not view the system accuracy x stance interaction or the trust in AI x stance interaction in Study 2 to be uniquely aligned with cognitive versus motivational reasoning. For example, epistemic motivations could enhance use of existing knowledge, not only pertaining to the general topic associated with one’s stance but also to the accuracy level of the fact-checking system or the trust in such systems that one has developed over repeated exposure to such systems. Alternatively, one could think of the accuracy of the system or trust in such systems as muting impact of the directional (stance-consistent) motivated processes that create the stance-consistent patterns in post-correction misinformation endorsement. The impact of system accuracy or trust in the system could again reflect epistemic motives, but in this case as muting directionally motivated cognition as opposed to more epistemic use of existing knowledge. Methodological tools such as misattribution paradigms could help to determine whether discomfort in the face of correction plays a motivational role in the effects [31]. For instance, it could be that epistemic use of system trust or use of knowledge about system accuracy might offset the motivated impact of correction-induced discomfort. It remains for future research to use such tools or develop new ones to help researchers determine the extent to which the current patterns of judgment reflect mostly cognitive (yet biased) evaluation of the truth of the misinformation or of the qualities of the fact-checking system versus more motivationally rich mixes of cognitive and motivational mechanisms.

Both the influences of prior beliefs on misinformation endorsement and on post-correction system qualities might be most potent when the prior beliefs are relatively strong rather than weak [70]. That is, prior beliefs related to a topic perceived as important might be most likely to continue to influence misinformation endorsement following correction [62] and might also be most likely to influence post-correction perceptions of an automated fact-checking system. As examined in the attitude strength literature, however, there are many different aspects of attitudes associated with their strength, such as ambivalence, certainty, knowledge, and basis in morality or values [71,72]. Future research could build on previous work focused on issue importance to examine additional dimensions of the strength of prior beliefs that influence misinformation endorsement and perceptions of correction sources. In future research, it would also be interesting to examine whether effects of the misinformation itself on participant stances might play a role in post-correction misinformation endorsement. Such pre-correction mechanisms would not necessary explain the moderation by correction source (AI system) characteristics, but similar to the earlier comments about importance, they could serve as an important background factor that facilitates the pattern of effects.

Additionally, future work should delve deeper into the role of “trust in automation” in predicting perceived system accuracy and changing the impact of prior beliefs. We used a brief and previously untested measure of trust in AI as a fact-checker. It had good reliability (internal consistency) and related in reasonable ways with other theoretically-connected concepts (such as perceived reliability of the automated system – in both studies) as well as moderating relations between prior stances and post-correction endorsement of the misinformation. Thus, there are important senses in which the predictive validity of the trust in AI measure supports its appropriateness. That said, we would also acknowledge that a longer, more complete measure of trust in AI might be useful for future research. That is, the reasonable relations we found with the brief measure in the current research suggests that it could be worthwhile to take the time in future research to include a more complete measure of trust in AI that uses multiple items to assess each of the components we sought to capture in the brief measure (i.e., performance, process, and purpose [52,53]). Human factors practitioners have also conceptualized trust in automation as composed of dispositional, situational, and learned dimensions [73]. The present research did not index the extent to which trust was dispositional or situational or the extent to which that trust had developed over time, but these dimensions could be fruitfully addressed in future research. Future researchers could also benefit from exploring how these different facets interact with prior beliefs and perceived accuracy to predict continued misinformation endorsement.

A possible limitation with this research lies in the unrealistically high level of described accuracy of the high-accuracy automated system in Study 2 (i.e., 97%). It is possible that at least some participants did not believe the purported accuracy in that condition, and that might be part of the reason participant stance continued to influence perceptions of the system and endorsement of the misinformation post-correction. If so, we might have found stronger differences across conditions if we had used a high-accuracy description that was less extreme but more believed by participants. Such an outcome would not be assured, though, in that those people who believed that the high-accuracy system really was nearly perfect (i.e., 97% correct) would be expected to show stronger effects of the manipulation than people who fully believed in a level of system accuracy that was less extreme. Any participants who did not believe that a system could be 97% accurate and dismissed the description would have undermined the strength of the manipulation and, to some degree, could have threatened the level of construct validity in the manipulation. Any such lack of belief would make it less likely to find differences across conditions (in both perceived system reliability and in impact of prior stances on continued belief). To the degree that we did find differences across conditions, however, the obtained pattern suggests some level of belief that the high-accuracy system was, in fact, a more reliable system than the low-accuracy system. For example, the main effect of system accuracy in Study 2 showed that the system was perceived as more reliable (M = 4.99; SD = 1.34) in the 97% accurate condition than in the 67% accurate condition (M = 4.53; SD = 1.35; *p* < .001). Differences in perceived system reliability combined with random assignment of the accuracy conditions to participants enhances the extent to which one can identify the manipulation per se as the cause of differences between the conditions (i.e., internal validity [74]). We want to acknowledge, however, that the potential effects of the manipulation might have been diminished by at least some participants failing to view the system as being as reliable as the unrealistic 97% accuracy value might have implied. The fact that mean perceived reliability was not near the top of the scale might suggest that some fairly large proportion of participants did not fully believe the high-accuracy description (though they perceived system reliability as overall higher than participants in the low-accuracy condition). If perceivers were to view automated/AI systems as having nearly perfect accuracy, we believe that influences of prior stances could be further reduced.

It is possible that many demographic or psychological variables might further moderate the current pattern of results. For example, it seems likely that the education level of our research participants was relatively high, especially compared with the more general population. In Study 1, we were able to access education level for each of our participants, and 61% of participants had either an undergraduate or graduate/professional degree. However, when treating education level (<bachelor’s vs. bachelor’s or higher) as a potential moderator of the relation between participants’ stance on gun control and their post-correction belief in pro-gun-control misinformation, there was little evidence of such moderation [for the stance x education interaction, *t*(298) = −1.0, *p* = .32]. We did not measure education level in Study 2, but samples from Amazon’s Mechanical Turk (MTurk) represent similarly high levels of education. Consistent with that notion, over 60% of MTurk participants have been reported to have an undergraduate or graduate degree [75]. Despite the lack of direct evidence of moderation of stance relations with post-correction misinformation endorsement by education when no information was given about the automated system, it remains possible that differences in the effects of system accuracy might be more likely for samples with relatively high rather than low levels of education. It could be important, therefore, that future work more completely address education level as a potential limiting factor. Depending on the topic of the misinformation and fact-check, it could also be that other moderators (either related to topic relevance or to perceptions of sources related to that domain) might determine for whom or when the current pattern of results would hold. As another example, we limited participant recruitment to a US-based sample because we expected Americans to have relatively strong prior beliefs about gun control and guns/crime constitute a highly relevant topic in the US. Future research would benefit from addressing a broader international sample to increase generalizability, though it might be necessary to use different topics in order to have strong enough prior beliefs that they influence post-correction misinformation endorsement (cf. [62]).

## Conclusions

In an era defined by unprecedented access to information, assisting individual users in evaluating the validity of incoming claims is vital to prevent the spread of misinformed beliefs. Automated fact checking systems that can efficiently cross-reference sources and provide reliable evaluations are likely to be a critical tool given the vast magnitude of the information space. Understanding how trustworthy people perceived these systems to be, how this influences the persuasiveness of the systems, and what role prior beliefs play in this relation are critically important. Our data suggest that when automated fact checkers are perceived as less than fully accurate, as is often the case, people readily discredit corrections that contradict their prior beliefs. Effective strategies to address this concern might include providing information about the system that bolsters perceived accuracy or shifting attention to factors other than system accuracy when people evaluate corrections. This research offers a novel first step in identifying the unique challenges faced by designers of scalable misinformation correction systems.

## Supporting information

S1 File PLOSONE Supplemental Analyses. (DOCX)

## References

[1] Chan M-PS, Albarracín D. A meta-analysis of correction effects in science-relevant misinformation. Nat Hum Behav. 2023;7(9):1514–25. doi: 10.1038/s41562-023-01623-8 37322236 PMC12397989

[2] Fridkin K, Kenney PJ, Wintersieck A. Liar, Liar, Pants on Fire: How Fact-Checking Influences Citizens’ Reactions to Negative Advertising. Political Communication. 2015;32(1):127–51. doi: 10.1080/10584609.2014.914613

[3] Garrett RK, Weeks BE. The promise and peril of real-time corrections to political misperceptions. In: Proceedings of the 2013 conference on Computer supported cooperative work. 2013;1047–58. doi: 10.1145/2441776.2441895

[4] Nyhan B, Reifler J. When corrections fail: the persistence of political misperceptions. Polit Behav. 2010;32(2):303–30. doi: 10.1007/s11109-010-9112-2

[5] Wood T, Porter E. The elusive backfire effect: mass attitudes’ steadfast factual adherence. Polit Behav. 2018;41(1):135–63. doi: 10.1007/s11109-018-9443-y

[6] Nyhan B. Why the backfire effect does not explain the durability of political misperceptions. Proc Natl Acad Sci U S A. 2021;118(15):e1912440117. doi: 10.1073/pnas.1912440117 33837144 PMC8053951

[7] Walter N, Murphy ST. How to unring the bell: A meta-analytic approach to correction of misinformation. Communication Monographs. 2018;85(3):423–41. doi: 10.1080/03637751.2018.1467564

[8] Hassan N, Li C, Tremayne M. Detecting check-worthy factual claims in presidential debates. in: proceedings of the 24th ACM international on conference on information and knowledge management. 2015;1835–8. doi: 10.1145/2806416.2806652

[9] Adair-Hinojosa C, Alsmadi I. Fake news detection and machine learning, ethical issues: a systematic literature review. SSRN Journal. 2022. doi: 10.2139/ssrn.4309139

[10] Lee J, Bissell K. User agency–based versus machine agency–based misinformation interventions: The effects of commenting and AI fact-checking labeling on attitudes toward the COVID-19 vaccination. New Media & Society. 2023;26(12):6817–37. doi: 10.1177/14614448231163228

[11] Vedula N, Parthasarathy S. FACE-KEG: fact checking explained using knowledge graphs. in: proceedings of the 14th ACM international conference on web search and data mining. 2021. 526–34. doi: 10.1145/3437963.3441828

[12] Vraga EK, Bode L. Correction as a solution for health misinformation on social media. Am J Public Health. 2020;110(S3):S278–80. doi: 10.2105/AJPH.2020.305916 33001724 PMC7532323

[13] Johnson HM, Seifert CM. Sources of the continued influence effect: When misinformation in memory affects later inferences. J Experimental Psychol: Learning, Memory, and Cognition. 1994;20(6):1420–36. doi: 10.1037/0278-7393.20.6.1420

[14] Anderson CA, Lepper MR, Ross L. Perseverance of social theories: The role of explanation in the persistence of discredited information. Journal of Personality and Social Psychology. 1980;39(6):1037–49. doi: 10.1037/h0077720

[15] Petty RE, Wegener DT, Fabrigar LR. Attitudes and attitude change. Annu Rev Psychol. 1997;48:609–47. doi: 10.1146/annurev.psych.48.1.609 9046570

[16] Schul Y, Burnstein E. When discounting fails: Conditions under which individuals use discredited information in making a judgment. J Personality Social Psychol. 1985;49(4):894–903. doi: 10.1037/0022-3514.49.4.894

[17] Ecker UKH, Lewandowsky S, Cook J, Schmid P, Fazio LK, Brashier N, et al. The psychological drivers of misinformation belief and its resistance to correction. Nat Rev Psychol. 2022;1(1):13–29. doi: 10.1038/s44159-021-00006-y

[18] Bond RM, Garrett RK. Engagement with fact-checked posts on Reddit. PNAS Nexus. 2023;2(3):pgad018. doi: 10.1093/pnasnexus/pgad018 36926223 PMC10011804

[19] Friggeri A, Adamic L, Eckles D, Cheng J. Rumor cascades. ICWSM. 2014;8(1):101–10. doi: 10.1609/icwsm.v8i1.14559

[20] Walter N, Cohen J, Holbert RL, Morag Y. Fact-checking: a meta-analysis of what works and for whom. Political Communication. 2019;37(3):350–75. doi: 10.1080/10584609.2019.1668894

[21] Lewandowsky S, Ecker UKH, Seifert CM, Schwarz N, Cook J. Misinformation and Its Correction: Continued Influence and Successful Debiasing. Psychol Sci Public Interest. 2012;13(3):106–31. doi: 10.1177/1529100612451018 26173286

[22] Heine SJ, Proulx T, Vohs KD. The meaning maintenance model: on the coherence of social motivations. Pers Soc Psychol Rev. 2006;10(2):88–110. doi: 10.1207/s15327957pspr1002_1 16768649

[23] Proulx T, Inzlicht M, Harmon-Jones E. Understanding all inconsistency compensation as a palliative response to violated expectations. Trends Cogn Sci. 2012;16(5):285–91. doi: 10.1016/j.tics.2012.04.002 22516239

[24] Weary G, Edwards JA. Causal-uncertainty beliefs and related goal structures. In: Sorrentino RM, Higgins ET, editors. Handbook of motivation and cognition. New York: The Guilford Press. 1996. p. 148–81.

[25] Kruglanski AW, Jasko K, Friston K. All Thinking is “Wishful” Thinking. Trends Cogn Sci. 2020;24(6):413–24. doi: 10.1016/j.tics.2020.03.004 32284177

[26] Kunda Z. The case for motivated reasoning. Psychol Bull. 1990;108(3):480–98. doi: 10.1037/0033-2909.108.3.480 2270237

[27] Taber CS, Lodge M. Motivated skepticism in the evaluation of political beliefs. American J Political Sci. 2006;50(3):755–69. doi: 10.1111/j.1540-5907.2006.00214.x

[28] Lodge M, Taber CS. The Rationalizing Voter. Cambridge University Press. 2013. doi: 10.1017/cbo9781139032490

[29] Edwards K, Smith EE. A disconfirmation bias in the evaluation of arguments. J Personality Social Psychol. 1996;71(1):5–24. doi: 10.1037/0022-3514.71.1.5

[30] Ecker UKH, Ang LC. Political attitudes and the processing of misinformation corrections. Political Psychol. 2018;40(2):241–60. doi: 10.1111/pops.12494

[31] Susmann MW, Wegener DT. How attitudes impact the continued influence effect of misinformation: the mediating role of discomfort. Pers Soc Psychol Bull. 2023;49(5):744–57. doi: 10.1177/01461672221077519 35227114

[32] Susmann MW, Wegener DT. The role of discomfort in the continued influence effect of misinformation. Mem Cognit. 2022;50(2):435–48. doi: 10.3758/s13421-021-01232-8 34533754 PMC8447889

[33] Swift A. Americans’ trust in mass media sinks to new low. http://www.gallup.com/poll/195542/americans-trust-mass-media-sinks-new-low.aspx. 2016.

[34] Shin J, Thorson K. Partisan selective sharing: the biased diffusion of fact-checking messages on social media. J Commun. 2017;67(2):233–55. doi: 10.1111/jcom.12284

[35] Barberá P, Jost JT, Nagler J, Tucker JA, Bonneau R. Tweeting from left to right. Psychol Sci. 2015;26(10):1531–42. doi: 10.1177/0956797615594620 26297377

[36] Guess A, Nagler J, Tucker J. Less than you think: prevalence and predictors of fake news dissemination on Facebook. Sci Adv. 2019;5(1):eaau4586. doi: 10.1126/sciadv.aau4586 30662946 PMC6326755

[37] González-Bailón S, Lazer D, Barberá P, Zhang M, Allcott H, Brown T, et al. Asymmetric ideological segregation in exposure to political news on Facebook. Science. 2023;381(6656):392–8. doi: 10.1126/science.ade7138 37499003

[38] Liu X, Qi L, Wang L, Metzger MJ. Checking the Fact-Checkers: The Role of Source Type, Perceived Credibility, and Individual Differences in Fact-Checking Effectiveness. Communication Res. 2023;52(6):719–46. doi: 10.1177/00936502231206419

[39] McGuire WJ. The Nature of Attitudes and Attitude Change. In: Lindzey G, Aronson E, editors. Handbook of Social Psychology. 3rd ed. New York: Random House. 1985. p. 233–346.

[40] Petty RE, Wegener DT. Attitude change: Multiple roles for persuasion variables. In: Gilbert DT, Fiske ST, Lindzey G, editors. The handbook of social psychology. 4th ed. McGraw-Hill. 1998. p. 323–90.

[41] Wallace LE, Wegener DT, Petty RE. When sources honestly provide their biased opinion: bias as a distinct source perception with independent effects on credibility and persuasion. Pers Soc Psychol Bull. 2020;46(3):439–53. doi: 10.1177/0146167219858654 31282841

[42] Wallace LE, Hinsenkamp L, Wegener DT, Braun Z. Effects of message-sidedness on perceived source bias: when presenting two sides does versus does not alleviate concerns about bias. Pers Soc Psychol Bull. 2024;50(5):807–20. doi: 10.1177/01461672231155389 36803257

[43] Hovland CI, Weiss W. The Influence of source credibility on communication effectiveness. Public Opinion Quarterly. 1951;15(4):635. doi: 10.1086/266350

[44] Luo M, Hancock JT, Markowitz DM. Credibility perceptions and detection accuracy of fake news headlines on social media: effects of truth-bias and endorsement cues. Communication Res. 2020;49(2):171–95. doi: 10.1177/0093650220921321

[45] Pornpitakpan C. The persuasiveness of source credibility: a critical review of five decades’ evidence. J Applied Social Pyschol. 2004;34(2):243–81. doi: 10.1111/j.1559-1816.2004.tb02547.x

[46] Yoon K, Kim CH, Kim M-S. A cross-cultural comparison of the effects of source credibility on attitudes and behavioral intentions. Mass Communication and Society. 1998;1(3–4):153–73. doi: 10.1080/15205436.1998.9677854

[47] Bruns H, Dessart FJ, Krawczyk M, Lewandowsky S, Pantazi M, Pennycook G, et al. Investigating the role of source and source trust in prebunks and debunks of misinformation in online experiments across four EU countries. Sci Rep. 2024;14(1):20723. doi: 10.1038/s41598-024-71599-6 39237648 PMC11377563

[48] Ecker UKH, Antonio LM. Can you believe it? An investigation into the impact of retraction source credibility on the continued influence effect. Mem Cognit. 2021;49(4):631–44. doi: 10.3758/s13421-020-01129-y 33452666 PMC7810102

[49] Susmann MW, Wegener DT. The independent effects of source expertise and trustworthiness on retraction believability: The moderating role of vested interest. Mem Cognit. 2023;51(4):845–61. doi: 10.3758/s13421-022-01374-3 36460863 PMC9718466

[50] Jia C, Lee T. Journalistic interventions matter: understanding how Americans perceive fact-checking labels. HKS Misinfo Rev. 2024. doi: 10.37016/mr-2020-138

[51] Lee JD, See KA. Trust in automation: designing for appropriate reliance. Hum Factors. 2004;46(1):50–80. doi: 10.1518/hfes.46.1.50_30392 15151155

[52] Kohn SC, de Visser EJ, Wiese E, Lee Y-C, Shaw TH. Measurement of trust in automation: a narrative review and reference guide. Front Psychol. 2021;12:604977. doi: 10.3389/fpsyg.2021.604977 34737716 PMC8562383

[53] Lee J, Moray N. Trust, control strategies and allocation of function in human-machine systems. Ergonomics. 1992;35(10):1243–70. doi: 10.1080/00140139208967392 1516577

[54] Merritt SM, Ilgen DR. Not all trust is created equal: dispositional and history-based trust in human-automation interactions. Hum Factors. 2008;50(2):194–210. doi: 10.1518/001872008X288574 18516832

[55] Askarisichani O, Bullo F, Friedkin NE, Singh AK. Predictive models for human-AI nexus in group decision making. Ann N Y Acad Sci. 2022;1514(1):70–81. doi: 10.1111/nyas.14783 35581156

[56] Ault MK, Ness AM, Taylor WD, Johnson G, Connelly S, Jensen ML, et al. Ideological lens matters: Credibility heuristics, pre-existing attitudes, and reactions to messages on ideological websites. Computers in Human Behavior. 2017;68:315–25. doi: 10.1016/j.chb.2016.11.053

[57] Chaiken S. The heuristic model of persuasion. In: Zanna MP, Olson JM, Herman CP, editors. Social influence: The Ontario symposium. Hillsdale, NJ: Lawrence Erlbaum Associates. 1987. p. 3–39.

[58] liberman A, Chaiken S. Defensive processing of personally relevant health messages. Pers Soc Psychol Bull. 1992;18(6):669–79. doi: 10.1177/0146167292186002

[59] Jia C, Boltz A, Zhang A, Chen A, Lee MK. Understanding Effects of algorithmic vs. community label on perceived accuracy of hyper-partisan misinformation. Proc ACM Hum-Comput Interact. 2022;6(CSCW2):1–27. doi: 10.1145/355509637360538

[60] Sundar SS, Kim J. Machine Heuristic. In: Proceedings of the 2019 CHI Conference on Human Factors in Computing Systems. 2019;1–9. doi: 10.1145/3290605.3300768

[61] Yaqub W, Kakhidze O, Brockman ML, Memon N, Patil S. Effects of Credibility Indicators on Social Media News Sharing Intent. In: Proceedings of the 2020 CHI Conference on Human Factors in Computing Systems. 2020. 1–14. doi: 10.1145/3313831.3376213

[62] Vidigal R, Jerit J. Issue Importance and the correction of misinformation. Political Communication. 2022;39(6):715–36. doi: 10.1080/10584609.2022.2123580

[63] Westbrook V, Wegener DT, Susmann MW. Mechanisms in continued influence: The impact of misinformation corrections on source perceptions. Mem Cognit. 2023;51(6):1317–30. doi: 10.3758/s13421-023-01402-w 36988856

[64] Ferrando PJ, Lorenzo-Seva U. Assessing the quality and appropriateness of factor solutions and factor score estimates in exploratory item factor analysis. Educ Psychol Meas. 2018;78(5):762–80. doi: 10.1177/0013164417719308 32655169 PMC7328234

[65] Gorsuch RL. Factor analysis: Classic edition. Routledge. 2014.

[66] Pew Research Center. Gun Violence Widely Viewed as a Major – and Growing – National Problem. https://www.pewresearch.org/politics/2023/06/28/gun-violence-widely-viewed-as-a-major-and-growing-national-problem/. 2023.

[67] Riedel B, Augenstein I, Spithourakis GP, Riedel S. A simple but tough-to-beat baseline for the fake news challenge stance detection task. arXiv preprint. 2017. doi: 10.48550/arXiv:1707.03264

[68] Pretus C, Servin-Barthet C, Harris EA, Brady WJ, Vilarroya O, Van Bavel JJ. The role of political devotion in sharing partisan misinformation and resistance to fact-checking. J Exp Psychol Gen. 2023;152(11):3116–34. doi: 10.1037/xge0001436 37347911

[69] Bullock JG. Partisan bias and the bayesian ideal in the study of public opinion. J Politics. 2009;71(3):1109–24. doi: 10.1017/s0022381609090914

[70] Petty RE, Krosnick JA. Attitude strength: antecedents and consequences. Hillsdale, NJ: Lawrence Erlbaum Associates. 1995.

[71] Philipp-Muller AZ, Wallace LE, Wegener DT. Where does moral conviction fit?: A factor analytic approach examining antecedents to attitude strength. J Experimental Social Psychol. 2020;86:103900. doi: 10.1016/j.jesp.2019.103900

[72] Wegener DT, Downing J, Krosnick JA, Petty RE. Strength-related properties of attitudes: Measures, manipulations, and future directions. In: Petty RE, Krosnick JA, editors. Attitude strength: Antecedents and consequences. Mahwah, NJ: Erlbaum. 1995. p. 455–87.

[73] Marsh S, Dibben MR. The role of trust in information science and technology. Annu Rev Inf Sci Technol. 2003;37:465–98.

[74] Shadish W, Cook TD, Campbell DT. Experimental and quasi-experimental designs for generalized causal inference. Boston, MA: Houghton Mifflin. 2002.

[75] Hauser D, Paolacci G, Chandler J. Common concerns with MTurk as a Participant Pool. Handbook of Research Methods in Consumer Psychology. Routledge. 2019. p. 319–37. doi: 10.4324/9781351137713-17

