# Supplementary material for: Prior beliefs & automated fact checking: Limits on the effectiveness of AI-based corrections
Source: PLoS One. 2026 Feb 5;21(2):e0342332. doi: 10.1371/journal.pone.0342332 (PMC12875456; doi:10.1371/journal.pone.0342332)
Supplement: S1 File — (DOCX) [file pone.0342332.s001.docx]

**Supplemental Analyses**

**Study 1**

The following sections go beyond the analyses reported in the main text by replacing the stance predictor variable with either gun control attitudes or with political ideology.

**Default (Uninformed) Accuracy Estimates**

When the regression model contained only gun control attitudes and trust in AI, both attitudes (*b* = 1.25, *t*[299] = 2.53, *p* = .012, *r* = .14) and trust in AI (*b* = 6.02, *t*[298] = 8.98, *p* < .001, *r* = .46) were significant predictors of participant accuracy predictions.

For Study 1, the descriptive statistics for political ideology are as follows: (*M* = 3.32, *SD* = 1.646, variance = 2.710, range = 6).


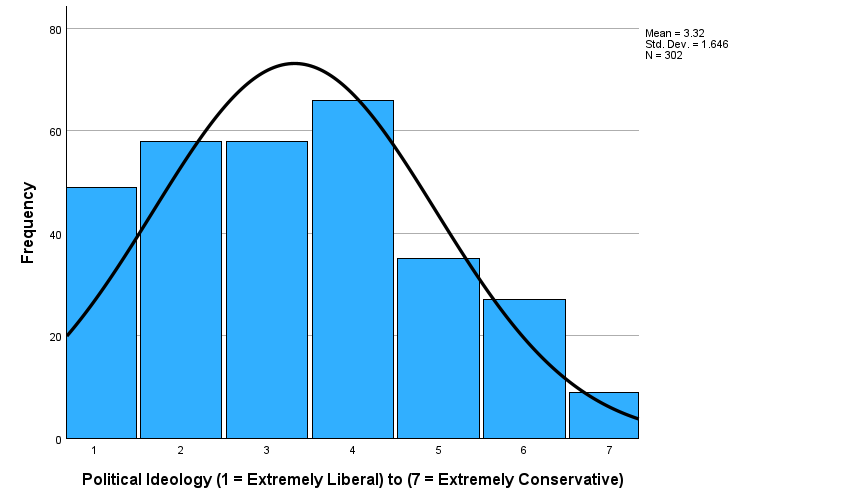


Of note, ideology was left in the original scale in analyses presented in this supplement. In the main text, political ideology was reverse-scored in order to combine it with attitudes in the stance variable. Thus, consistent results for attitudes and ideology in this supplement will have opposite signs. When the regression model contained only political ideology and Trust in AI, both political ideology (*b* = -1.46, *t*[299] = -2.45, *p* = .015, *r* = .14) and Trust in AI (*b* = 6.33, *t*[298] = 9.58, *p* < .001, *r* = .49) were significant predictors of participant accuracy predictions.

**Post-correction Misinformation Endorsement**

When gun control attitudes were entered in the regression model alongside AI trust predicting misinformation endorsement, AI trust was non-significant and attitudes remained significant, *b* = .29, *t*(299) = 5.82, *p* < .001, *r* = .32. When political ideology was entered in the regression model alongside AI trust, AI trust was not significant, but political ideology bordered on significance, *b* = -.12, *t*(299) = -1.94, *p* = .053, *r* = .11.

**Post-correction Reliability Perceptions of Automated Fact-Checker**

We conducted a multiple regression predicting post-correction reliability perceptions using trust in AI and participant political ideology as predictor variables. Trust in AI significantly predicted post-correction reliability perceptions, *b* = .47, *t*(299) = 9.83, *p <* .001, *r* = .49, but participant political ideology did not, *b* = .03, *t*(299) = .687, *p =* .49, *r* = .007.

We conducted a multiple regression predicting post-correction reliability perceptions using trust in AI and participant attitudes as predictor variables. Trust in AI significantly predicted post-correction reliability perceptions, *b* = .47, *t*(299) = 9.697, *p <* .001, *r* = .49, but participant gun control attitudes did not, *b* = -.004, *t*(299) = -.12, *p =* .90, *r* = .007.

**Post-correction Bias Perceptions of Automated Fact-Checker**

We conducted a multiple regression predicting post-correction bias perceptions of the automated fact checker using trust in AI and participant gun control attitudes as predictor variables. Trust in AI significantly predicted post-correction bias perceptions, *b* = -.25*, t*[299] = -4.99, *p* < .001, *r* = .28, but participant gun control attitudes did not, *b* = -.014*, t*[299] = .390, *p* = .70, *r* = .023.

We conducted a multiple regression predicting post-correction bias perceptions of the automated fact checker using trust in AI and participant political ideology as predictor variables. Trust in AI significantly predicted post-correction bias perceptions, *b* = -.26*, t*[299] = -5.16, *p* < .001, *r* = .29, but participant political ideology did not, *b* = .056 *t*[299] = 1.26, *p* = .21, *r* = .07

**Study 2**

As indicated in the main text, we conducted a general linear model (regression) analysis that included the following terms as centered predictors of post-correction misinformation endorsement: accuracy condition, stance, trust in AI, type of accuracy manipulation and all interactions. Table S1 shows the statistics for all relations, including ones omitted from the main text.

**Table S1**

*Accuracy Condition, Trust in AI, Manipulation Type, and Stance Predicting Misinformation Endorsement*

| **Predictor Variable** | ***B*** | **Mean Square** | **full model error term** | ***F*** | ***t*** | **Error df full model** | ***p*** |
| --- | --- | --- | --- | --- | --- | --- | --- |
| Accuracy Condition | -0.51 | 68.87 | 2.93 | 23.50 | 4.85 | 1055 | <.001 |
| Trust in AI | -0.02 | 0.90 | 2.93 | 0.31 | 0.55 | 1055 | 0.58 |
| Manipulation Type | -0.62 | 70.78 | 2.93 | 24.15 | 4.91 | 1055 | <.001 |
| Stance | 0.35 | 385.49 | 2.93 | 131.52 | 11.47 | 1055 | <.001 |
| Trust in AI x Stance | -0.08 | 41.24 | 2.93 | 14.07 | 3.75 | 1055 | <.001 |
| Accuracy Condition x Stance | -0.15 | 17.00 | 2.93 | 5.80 | 2.41 | 1055 | .02 |
| Manipulation Type x Stance | 0.07 | 2.80 | 2.93 | 0.96 | 0.98 | 1055 | 0.33 |
| Accuracy Condition x Trust in AI | -0.09 | 3.56 | 2.93 | 1.21 | 1.10 | 1055 | 0.27 |
| Trust in AI x Manipulation Type | -0.51 | 88.17 | 2.93 | 30.08 | 5.48 | 1055 | <.001 |
| Accuracy Condition x Manipulation Type | 0.19 | 1.58 | 2.93 | 0.54 | 0.73 | 1055 | 0.46 |
| Accuracy Condition x Trust in AI x Stance | -0.04 | 2.67 | 2.93 | 0.91 | 0.95 | 1055 | 0.34 |
| Trust in AI x Manipulation Type x Stance | 0.11 | 13.33 | 2.93 | 4.55 | 2.13 | 1055 | 0.03 |
| Accuracy Condition x Manipulation Type x Stance | 0.05 | 0.72 | 2.93 | 0.24 | 0.49 | 1055 | 0.62 |
| Accuracy Condition x Trust in AI x Manipulation Type | 0.17 | 2.43 | 2.93 | 0.83 | 0.91 | 1055 | 0.36 |
| Accuracy Condition x Trust in AI x Manipulation Type x Stance | 0.13 | 4.60 | 2.93 | 1.57 | 1.25 | 1055 | 0.21 |

As indicated in the main text, we also conducted the same analyses as above but used gun control attitudes as a predictor in the model instead of stance, as shown in Table S2.

**Table S2**

*Accuracy Condition, Trust in AI, Manipulation Type, and Gun Control Attitudes Predicting Misinformation Endorsement*

| **Predictor Variable** | **B** | **Mean Square** | **full model error term** | **F** | **t** | **df full model** | **p** |
| --- | --- | --- | --- | --- | --- | --- | --- |
| Accuracy Condition | -0.51 | 69.58 | 2.82 | 24.70 | 4.97 | 1055 | <.001 |
| Trust in AI | -0.08 | 10.45 | 2.82 | 3.71 | 1.93 | 1055 | 0.05 |
| Manipulation Type | -0.58 | 61.53 | 2.82 | 21.84 | 4.67 | 1055 | <.001 |
| Attitudes | 0.36 | 583.66 | 2.82 | 207.19 | 14.39 | 1055 | <.001 |
| Trust in AI x Attitudes | -0.02 | 5.17 | 2.82 | 1.83 | 1.35 | 1055 | 0.18 |
| Accuracy Condition x Attitudes | -0.12 | 15.83 | 2.82 | 5.62 | 2.37 | 1055 | 0.02 |
| Manipulation Type x Attitudes | -0.01 | 0.06 | 2.82 | 0.02 | 0.14 | 1055 | 0.88 |
| Accuracy Condition x Trust in AI | -0.09 | 2.49 | 2.82 | 0.88 | 0.94 | 1055 | 0.35 |
| Trust in AI x Manipulation Type | -0.45 | 66.89 | 2.82 | 23.75 | 4.87 | 1055 | <.001 |
| Accuracy Condition x Manipulation Type | 0.15 | 1.07 | 2.82 | 0.38 | 0.61 | 1055 | 0.54 |
| Accuracy Condition x Trust in AI x Attitudes | -0.06 | 7.60 | 2.82 | 2.70 | 1.64 | 1055 | 0.10 |
| Trust in AI x Manipulation Type x Attitudes | 0.04 | 2.02 | 2.82 | 0.72 | 0.85 | 1055 | 0.40 |
| Accuracy Condition x Manipulation Type x Attitudes | 0.03 | 0.15 | 2.82 | 0.05 | 0.23 | 1055 | 0.82 |
| Accuracy Condition x Trust in AI x Manipulation Type | 0.12 | 1.17 | 2.82 | 0.42 | 0.64 | 1055 | 0.52 |
| Accuracy Condition x Trust in AI x Manipulation Type x Attitudes | 0.03 | 1.49 | 2.82 | 0.53 | 0.73 | 1055 | 0.47 |

As indicated in the main text, we also conducted the same analyses as above but used political ideology as a predictor in the model instead of stance, as shown in Table S2. Figure S2 shows the distribution of political ideology found in the sample.

**Table S3**

*Accuracy Condition, Trust in AI, Manipulation Type, and Political Ideology Predicting Misinformation Endorsement*

| **Predictor Variable** | **B** | **Mean Square** | **full model error term** | **F** | **t** | **df full model** | **p** |
| --- | --- | --- | --- | --- | --- | --- | --- |
| Accuracy Condition | -0.51 | 69.72 | 3.13 | 22.27 | 4.72 | 1055 | <.001 |
| Trust in AI | 0.03 | 1.31 | 3.13 | 0.42 | 0.65 | 1055 | 0.52 |
| Manipulation Type | -0.61 | 67.97 | 3.13 | 21.71 | 4.66 | 1055 | <.001 |
| Political Ideology | -0.17 | 96.08 | 3.13 | 30.69 | 5.54 | 1055 | <.001 |
| Trust in AI x Political Ideology | 0.13 | 120.10 | 3.13 | 38.36 | 6.19 | 1055 | <.001 |
| Accuracy Condition x Political Ideology | 0.11 | 10.90 | 3.13 | 3.48 | 1.87 | 1055 | 0.06 |
| Manipulation Type x Political Ideology | -0.05 | 1.74 | 3.13 | 0.56 | 0.75 | 1055 | 0.46 |
| Accuracy Condition x Trust in AI | -0.12 | 6.01 | 3.13 | 1.92 | 1.38 | 1055 | 0.17 |
| Trust in AI x Manipulation Type | -0.47 | 73.06 | 3.13 | 23.34 | 4.83 | 1055 | <.001 |
| Accuracy Condition x Manipulation Type | 0.15 | 1.03 | 3.13 | 0.33 | 0.57 | 1055 | 0.57 |
| Accuracy Condition x Trust in AI x Political Ideology | -0.01 | 0.20 | 3.13 | 0.06 | 0.25 | 1055 | 0.80 |
| Trust in AI x Manipulation Type x Political Ideology | -0.09 | 10.99 | 3.13 | 3.51 | 1.87 | 1055 | 0.06 |
| Accuracy Condition x Manipulation Type x Political Ideology | -0.08 | 0.95 | 3.13 | 0.30 | 0.55 | 1055 | 0.58 |
| Accuracy Condition x Trust in AI x Manipulation Type | 0.18 | 2.55 | 3.13 | 0.82 | 0.90 | 1055 | 0.37 |
| Accuracy Condition x Trust in AI x Manipulation Type x Political Ideology | -0.12 | 5.18 | 3.13 | 1.65 | 1.29 | 1055 | 0.20 |

For study 2, the descriptive statistics for political ideology are as follows: (*M* = 3.70, *SD* = 1.825, variance = 3.329, range = 6).

**Figure S2**

*Study 2 Political Ideology Distribution*


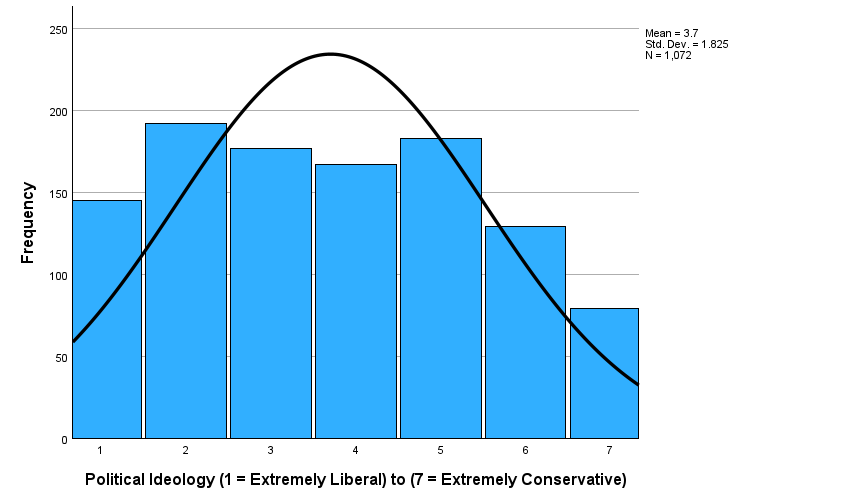


**Analyses Predicting Misinformation Endorsement Without Manipulation Type as a Factor**

***Post-Correction Misinformation Endorsement with Stance***

We conducted a regression analysis that included the following terms as centered predictors of post-correction misinformation endorsement: accuracy condition, stance, trust in AI, and all interactions. There was a significant main effect of accuracy condition such that corrections by a more accurate system resulted in less post-correction endorsement of the misinformation, *b* = -.52, *t*(1063) = -4.78, *p* < .001, *r* = .15. There was also a significant main effect of stance such that participants who were relatively liberal and/or more supportive of gun control continued to endorse the pro-gun-control misinformation after the correction more than those who were relatively conservative and/or against gun-control, *b* = .32, *t*(1063) = 10.12, *p* < .001, *r* = .30. These main effects were only significant when we included an interaction between the two terms (condition x stance), *b* = -.136, *t*(1063) = -2.15, *p* = .032, *r* = .07, as shown in *Figure 1*. Stance was more strongly related to post-correction misinformation endorsement when system accuracy was manipulated to be low, *b* = .39, *t*(1063) = 8.92, *p* < .001, *r* = .26, than when system accuracy was manipulated to be high, *b* = .25, *t*(1063) = 5.48, *p* < .001, *r* = .17. Viewed another way, for those who were relatively conservative and/or unsupportive of gun control (-1 *SD*), the manipulation of the accuracy did not have a significant effect on their post-correction misinformation endorsement, *b* = -.28, *t*(1063) = -1.81, *p* = .071, *r* = .06. In contrast, for those who were relatively liberal and/or supportive of gun control (+1 *SD*), the purported accuracy of the system affected their post-correction misinformation endorsement such that the more accurate system led to less post-correction misinformation endorsement, *b* = -.75, *t*(1063) = -4.92, *p* < .001, *r* = .15.

As with the post-correction misinformation endorsement measure in Study 1, no main effect of trust in AI was found. However, as suggested by the notion of correction source fallibility, a significant two-way interaction occurred between trust in AI and stance, *b* = -.09, *t*(1063) = -4.12, *p* < .001, *r* = .13, as shown in *Figure 2*. Holding a more liberal stance predicted endorsement of the pro-gun control misinformation more strongly when trust in AI was relatively low (-1 *SD*), *b* = .44, *t*(1063) = 11.20, *p* < .001, *r* = .32, compared to high (+1 *SD*), *b* = .20, *t*(1063) = 4.25, *p* < .001, *r* = .13. In addition, for participants with a relatively conservative, anti-gun control stance (-1 *SD*), misinformation endorsement was greater among those with higher levels of trust in AI, *b* = .16, *t*(1063) = 2.96, *p* = .003, *r* = .09. However, the opposite relation emerged among participants who held relatively liberal, pro-gun control views—endorsement of the misinformation increased as trust in AI decreased (*b* = -.15, *t*[1063] = -2.68, *p* = .007, *r* = .08). No additional effects approached significance *(ps* ≥ .14).

***Post-Correction Misinformation Endorsement with Gun Control Attitudes Rather than Stance***

We conducted a regression analysis that included the following terms as centered predictors of post-correction misinformation endorsement: accuracy condition, gun control attitudes, trust in AI, and all interactions. There was a significant main effect of accuracy condition such that corrections by a more accurate system resulted in less post-correction endorsement of the misinformation, *b* = -.50, *t*(1063) = -4.67, *p* < .001, *r* = .14. There was also a significant main effect of gun control attitudes such that participants who were more supportive of gun control continued to endorse the pro-gun-control misinformation after the correction more than those who were relatively conservative and/or against gun-control, *b* = .35, *t*(1063) = 13.55, *p* < .001, *r* = .38. These main effects were only significant when we included an interaction between the two terms (condition x attitudes), *b* = -.13, *t*(1063) = -2.50, *p* = .013, *r* = .08. No other main effects or interactions were found to be significant significance *(ps* ≥ .21).

***Post-Correction Misinformation Endorsement with Political Ideology Rather than Stance***

We conducted a regression analysis that included the following terms as centered predictors of post-correction misinformation endorsement: accuracy condition, political ideology, trust in AI, and all interactions. There was a significant main effect of accuracy condition such that corrections by a more accurate system resulted in less post-correction endorsement of the misinformation, *b* = -.50, *t*(1064) = -4.55, *p* < .001, *r* = .14. There was also a significant main effect of political ideology such that participants who were relatively liberal continued to endorse the pro-gun-control misinformation after the correction more than those who were relatively conservative and/or against gun-control, *b* = -.162, *t*(1064) = -5.36, *p* < .001, *r* = .16.

As with the post-correction misinformation endorsement measure in Study 1, no main effect of trust in AI was found. However, as suggested by the notion of correction source fallibility, a significant two-way interaction occurred between trust in AI and political ideology, *b* = .144, *t*(1064) = 6.97, *p* < .001, *r* = .21. No additional effects approached significance *(ps* ≥ .12).

**Post-correction System Reliability Perceptions**

As indicated in the main text, we conducted a general linear model (regression) analysis that included the following terms as centered predictors of post-correction system reliability perceptions: accuracy condition, stance, trust in AI, type of accuracy manipulation and all interactions. Table S4 shows the statistics for all relations, including ones omitted from the main text.

**Table S4**

*Accuracy Condition, Trust in AI, Manipulation Type, and Stance Predicting Reliability Perception*

| **Predictor Variable** | **B** | **Mean Square** | **full model error term** | **F** | **t** | **Error df full model** | **p** |
| --- | --- | --- | --- | --- | --- | --- | --- |
| Accuracy Condition | 0.40 | 41.63 | 1.20 | 34.60 | 5.88 | 1055 | <.001 |
| Trust in AI | 0.57 | 591.04 | 1.20 | 491.31 | 22.17 | 1055 | <.001 |
| Manipulation Type | 0.02 | 0.05 | 1.20 | 0.04 | 0.20 | 1055 | 0.84 |
| Stance | -0.13 | 52.27 | 1.20 | 43.45 | 6.59 | 1055 | <.001 |
| Trust in AI x Stance | 0.00 | 0.09 | 1.20 | 0.07 | 0.27 | 1055 | 0.79 |
| Accuracy Condition x Stance | 0.09 | 6.66 | 1.20 | 5.54 | 2.35 | 1055 | 0.02 |
| Manipulation Type x Stance | 0.03 | 0.57 | 1.20 | 0.48 | 0.69 | 1055 | 0.49 |
| Accuracy Condition x Trust in AI | 0.09 | 3.88 | 1.20 | 3.22 | 1.80 | 1055 | 0.07 |
| Trust in AI x Manipulation Type | -0.07 | 1.50 | 1.20 | 1.25 | 1.12 | 1055 | 0.26 |
| Accuracy Condition x Manipulation Type | -0.03 | 0.04 | 1.20 | 0.03 | 0.18 | 1055 | 0.86 |
| Accuracy Condition x Trust in AI x Stance | -0.01 | 0.06 | 1.20 | 0.05 | 0.22 | 1055 | 0.82 |
| Trust in AI x Manipulation Type x Stance | -0.04 | 1.68 | 1.20 | 1.39 | 1.18 | 1055 | 0.24 |
| Accuracy Condition x Manipulation Type x Stance | 0.05 | 0.28 | 1.20 | 0.23 | 0.48 | 1055 | 0.63 |
| Accuracy Condition x Trust in AI x Manipulation Type | 0.13 | 1.40 | 1.20 | 1.16 | 1.08 | 1055 | 0.28 |
| Accuracy Condition x Trust in AI x Manipulation Type x Stance | 0.06 | 0.89 | 1.20 | 0.74 | 0.86 | 1054 | 0.39 |

As indicated in the main text, we also conducted the same analyses as above but used gun control attitudes as a predictor in the model instead of stance, as shown in Table S5

**Table S5**

*Accuracy Condition, Trust in AI, Manipulation Type, and Gun Control Attitudes Predicting Reliability Perception*

| **Predictor Variable** | **B** | **Mean Square** | **full model error term** | **F** | **t** | **df full model** | **p** |
| --- | --- | --- | --- | --- | --- | --- | --- |
| Accuracy Condition | 0.40 | 42.06 | 1.21 | 34.67 | 5.89 | 1055 | <.001 |
| Trust in AI | 0.26 | 598.75 | 1.21 | 493.61 | 22.22 | 1055 | <.001 |
| Manipulation Type | 0.00 | 0.00 | 1.21 | 0.00 | 0.00 | 1055 | 1.00 |
| Attitudes | -0.10 | 41.28 | 1.21 | 34.03 | 5.83 | 1055 | <.001 |
| Trust in AI x Attitudes | 0.00 | 0.12 | 1.21 | 0.10 | 0.32 | 1055 | 0.75 |
| Accuracy Condition x Attitudes | 0.05 | 3.18 | 1.21 | 2.62 | 1.62 | 1055 | 0.11 |
| Manipulation Type x Attitudes | 0.04 | 0.90 | 1.21 | 0.74 | 0.86 | 1055 | 0.39 |
| Accuracy Condition x Trust in AI | 0.09 | 3.20 | 1.21 | 2.64 | 1.62 | 1055 | 0.11 |
| Trust in AI x Manipulation Type | -0.10 | 3.15 | 1.21 | 2.60 | 1.61 | 1055 | 0.11 |
| Accuracy Condition x Manipulation Type | 0.00 | 0.00 | 1.21 | 0.00 | 0.00 | 1055 | 1.00 |
| Accuracy Condition x Trust in AI x Attitudes | -0.02 | 0.72 | 1.21 | 0.59 | 0.77 | 1055 | 0.44 |
| Trust in AI x Manipulation Type x Attitudes | -0.03 | 1.39 | 1.21 | 1.15 | 1.07 | 1055 | 0.28 |
| Accuracy Condition x Manipulation Type x Attitudes | 0.06 | 0.64 | 1.21 | 0.52 | 0.72 | 1055 | 0.47 |
| Accuracy Condition x Trust in AI x Manipulation Type | 0.14 | 1.54 | 1.21 | 1.27 | 1.13 | 1055 | 0.26 |
| Accuracy Condition x Trust in AI x Manipulation Type x Attitudes | 0.09 | 2.56 | 1.21 | 2.11 | 1.45 | 1055 | 0.15 |

As indicated in the main text, we also conducted the same analyses as above but used political ideology as a predictor in the model instead of stance, as shown in Table S6.

**Table S6**

*Accuracy Condition, Trust in AI, Manipulation Type, and Political Ideology Predicting Reliability Perception*

| **Predictor Variable** | **B** | **Mean Square** | **full model error term** | **F** | **t** | **df full model** | **p** |
| --- | --- | --- | --- | --- | --- | --- | --- |
| Accuracy Condition | 0.39 | 41.36 | 1.21 | 34.12 | 5.84 | 1056 | <.001 |
| Trust in AI | 0.55 | 558.10 | 1.21 | 460.48 | 21.46 | 1056 | <.001 |
| Manipulation Type | 0.03 | 0.12 | 1.21 | 0.09 | 0.31 | 1056 | 0.76 |
| Political Ideology | 0.11 | 40.78 | 1.21 | 33.65 | 5.80 | 1056 | <.001 |
| Trust in AI x Political Ideology | -0.02 | 1.63 | 1.21 | 1.34 | 1.16 | 1056 | 0.25 |
| Accuracy Condition x Political Ideology | -0.09 | 7.82 | 1.21 | 6.45 | 2.54 | 1056 | 0.01 |
| Manipulation Type x Political Ideology | -0.01 | 0.05 | 1.21 | 0.04 | 0.21 | 1056 | 0.83 |
| Accuracy Condition x Trust in AI | 0.11 | 5.30 | 1.21 | 4.37 | 2.09 | 1056 | 0.04 |
| Trust in AI x Manipulation Type | -0.05 | 0.91 | 1.21 | 0.75 | 0.87 | 1056 | 0.39 |
| Accuracy Condition x Manipulation Type | -0.05 | 0.10 | 1.21 | 0.08 | 0.28 | 1056 | 0.78 |
| Accuracy Condition x Trust in AI x Political Ideology | -0.02 | 0.00 | 1.21 | 0.00 | 0.05 | 1056 | 0.96 |
| Trust in AI x Manipulation Type x Political Ideology | 0.04 | 1.79 | 1.21 | 1.48 | 1.22 | 1056 | 0.22 |
| Accuracy Condition x Manipulation Type x Political Ideology | -0.03 | 0.10 | 1.21 | 0.09 | 0.29 | 1056 | 0.77 |
| Accuracy Condition x Trust in AI x Manipulation Type | 0.12 | 1.19 | 1.21 | 0.98 | 0.99 | 1056 | 0.32 |
| Accuracy Condition x Trust in AI x Manipulation Type x Political Ideology | -0.02 | 0.08 | 1.21 | 0.06 | 0.25 | 1056 | 0.80 |

***Post-correction System Reliability Perceptions Without Manipulation Type as a Factor***

The four post-correction reliability perception measures were combined to form an index (α = .94). We conducted a regression with centered terms for stance, accuracy condition, trust in AI, and their interactions predicting post-correction reliability perceptions. There was a significant main effect of the accuracy manipulation on post-correction reliability perceptions where those who encountered the high accuracy system rated it as more reliable than those who encountered the low accuracy system, *b* = .40, *t*(1063) = 5.90, *p* < .001, *r* = 18. There was also a significant main effect of stance on post-correction reliability perceptions such that participants who were more liberal and/or pro-gun-control viewed the system as less reliable than participants who were more conservative and/or anti-gun-control, *b* = -.13, *t*(1063) = -6.39, *p* < .001, *r* = .19. Thus, the system was perceived as more reliable when it aligned with what people wanted to hear rather than with their default perceptions of system quality. There was also a significant main effect of trust in AI on post-correction reliability perceptions where those who were more trusting in AI found the system to be more reliable, *b* = .570, *t*(1063) = 22.69, *p* < .001, *r* = .57. Lastly, there was a significant two-way interaction between stance and accuracy condition that paralleled the post-correction endorsement of misinformation, *b* = .092, *t*(1063) = 2.32, *p* = .020, *r* = .11. That is, stance was more strongly related to post-correction reliability perceptions for the low system accuracy condition *b* = -.17, *t*(534) = -6.15, *p* < .001, *r* = .26, than for the high system accuracy condition, *b* = -.08, *t (*529) = -2.87, *p* = .004, *r* = .12. Of note, an interaction between accuracy condition and trust in AI approached statistical significance, *b* = .09, *t*(1063) = 1.77, *p* = .077, *r* = .05, suggesting that perceived reliability might be more affected by accuracy condition when trust in AI was high rather than low.

***Post-correction System Reliability Perceptions with Gun Control Attitudes Rather than Stance***

The four post-correction reliability perception measures were combined to form an index (α = .94). We conducted a regression with centered terms for gun control attitudes, accuracy condition, trust in AI, and their interactions predicting post-correction reliability perceptions. There was a significant main effect of the accuracy manipulation on post-correction reliability perceptions where those who encountered the high accuracy system rated it as more reliable than those who encountered the low accuracy system, *b* = .401, *t*(1063) = 5.97, *p* < .001, *r* = 18. There was also a significant main effect of gun control attitudes on post-correction reliability perceptions such that participants who were more pro-gun-control viewed the system as less reliable than participants who were more anti-gun-control, *b* = -.092, *t*(1063) = -5.51, *p* < .001, *r* = .17. Thus, the system was perceived as more reliable when it aligned with what people wanted to hear rather than with their default perceptions of system quality. There was also a significant main effect of trust in AI on post-correction reliability perceptions where those who were more trusting in AI found the system to be more reliable, *b* = .58, *t*(1063) = 22.84, *p* < .001, *r* = .57. There were no significant interactions found (*ps* > .12).

***Post-correction System Reliability Perceptions with Political Ideology Rather than Stance***

The four post-correction reliability perception measures were combined to form an index (α = .94). We conducted a regression with centered terms for political ideology, accuracy condition, trust in AI, and their interactions predicting post-correction reliability perceptions. There was a significant main effect of the accuracy manipulation on post-correction reliability perceptions where those who encountered the high accuracy system rated it as more reliable than those who encountered the low accuracy system, *b* = .40, *t*(1064) = 5.82, *p* < .001, *r* = 18. There was also a significant main effect of political ideology on post-correction reliability perceptions such that participants who were more liberal viewed the system as less reliable than participants who were more conservative, *b* = .11, *t*(1064) = 5.86, *p* < .001, *r* = .18. Thus, the system was perceived as more reliable when it aligned with what people wanted to hear rather than with their default perceptions of system quality. There was also a significant main effect of trust in AI on post-correction reliability perceptions where those who were more trusting in AI found the system to be more reliable, *b* = .55, *t*(1064) = 21.96, *p* < .001, *r* = .56. Lastly, there was a significant two-way interaction between political ideology and accuracy condition that paralleled the post-correction endorsement of misinformation, *b* = -.096, *t*(1064) = -2.59, *p* = .010, *r* = .08. Additionally, a significant interaction was found between accuracy condition and trust in AI, *b* = .11, *t*(1064) = 2.14, *p* = .033, *r* = .07. There were no significant interactions found (*ps* > .25)

**Post-correction Bias Perceptions**

As indicated in the main text, we conducted a general linear model (regression) analysis that included the following terms as centered predictors of post-correction system bias perception: accuracy condition, stance, trust in AI, type of accuracy manipulation and all interactions. Table S7 shows the statistics for all relations, including ones omitted from the main text.

**Table S7**

*Accuracy Condition, Trust in AI, Manipulation Type, and Stance Predicting Bias Perception*

| **Predictor Variable** | **B** | **Mean Square** | **full model error term** | **F** | **t** | **df full model** | **p** |
| --- | --- | --- | --- | --- | --- | --- | --- |
| Accuracy Condition | -0.29 | 22.98 | 1.78 | 12.92 | 3.60 | 1054 | <.001 |
| Trust in AI | -0.27 | 130.81 | 1.78 | 73.57 | 8.58 | 1054 | <.001 |
| Manipulation Type | -0.43 | 34.13 | 1.78 | 19.20 | 4.38 | 1054 | <.001 |
| Stance | 0.04 | 5.80 | 1.78 | 3.26 | 1.81 | 1054 | 0.07 |
| Trust in AI x Stance | -0.04 | 10.58 | 1.78 | 5.95 | 2.44 | 1054 | 0.01 |
| Accuracy Condition x Stance | -0.06 | 2.57 | 1.78 | 1.44 | 1.20 | 1054 | 0.23 |
| Manipulation Type x Stance | 0.03 | 0.37 | 1.78 | 0.21 | 0.46 | 1054 | 0.65 |
| Accuracy Condition x Trust in AI | -0.21 | 20.74 | 1.78 | 11.66 | 3.42 | 1054 | <.001 |
| Trust in AI x Manipulation Type | -0.29 | 27.87 | 1.78 | 15.68 | 3.96 | 1054 | <.001 |
| Accuracy Condition x Manipulation Type | 0.05 | 0.09 | 1.78 | 0.05 | 0.23 | 1054 | 0.82 |
| Accuracy Condition x Trust in AI x Stance | 0.03 | 1.34 | 1.78 | 0.75 | 0.87 | 1054 | 0.39 |
| Trust in AI x Manipulation Type x Stance | 0.10 | 10.92 | 1.78 | 6.14 | 2.48 | 1054 | 0.01 |
| Accuracy Condition x Manipulation Type x Stance | 0.02 | 0.05 | 1.78 | 0.03 | 0.16 | 1054 | 0.87 |
| Accuracy Condition x Trust in AI x Manipulation Type | -0.09 | 0.58 | 1.78 | 0.33 | 0.57 | 1054 | 0.57 |
| Accuracy Condition x Trust in AI x Manipulation Type x Stance | 0.02 | 0.10 | 1.78 | 0.05 | 0.23 | 1054 | 0.82 |

As indicated in the main text, we also conducted the same analyses as above but used gun control attitudes as a predictor in the model instead of stance, as shown in Table S8.

**Table S8**

*Accuracy Condition, Trust in AI, Manipulation Type, and Gun Control Attitudes Predicting Bias Perception*

| **Predictor Variable** | **B** | **Mean Square** | **full model error term** | **F** | **t** | **df full model** | **p** |
| --- | --- | --- | --- | --- | --- | --- | --- |
| Accuracy Condition | -0.29 | 22.91 | 1.78 | 12.85 | 3.58 | 1054 | <.001 |
| Trust in AI | -0.28 | 141.83 | 1.78 | 79.54 | 8.92 | 1054 | <.001 |
| Manipulation Type | -0.43 | 33.52 | 1.78 | 18.80 | 4.34 | 1054 | <.001 |
| Attitudes | 0.07 | 22.51 | 1.78 | 12.62 | 3.55 | 1054 | <.001 |
| Trust in AI x Attitudes | 0.00 | 0.05 | 1.78 | 0.03 | 0.16 | 1054 | 0.87 |
| Accuracy Condition x Attitudes | -0.05 | 2.96 | 1.78 | 1.66 | 1.29 | 1054 | 0.20 |
| Manipulation Type x Attitudes | -0.06 | 2.66 | 1.78 | 1.49 | 1.22 | 1054 | 0.22 |
| Accuracy Condition x Trust in AI | -0.22 | 21.53 | 1.78 | 12.07 | 3.47 | 1054 | <.001 |
| Trust in AI x Manipulation Type | -0.26 | 21.79 | 1.78 | 12.22 | 3.50 | 1054 | <.001 |
| Accuracy Condition x Manipulation Type | 0.04 | 0.07 | 1.78 | 0.04 | 0.19 | 1054 | 0.85 |
| Accuracy Condition x Trust in AI x Attitudes | 0.01 | 0.44 | 1.78 | 0.25 | 0.50 | 1054 | 0.62 |
| Trust in AI x Manipulation Type x Attitudes | 0.01 | 0.03 | 1.78 | 0.02 | 0.14 | 1054 | 0.89 |
| Accuracy Condition x Manipulation Type x Attitudes | 0.00 | 0.00 | 1.78 | 0.00 | 0.00 | 1054 | 1.00 |
| Accuracy Condition x Trust in AI x Manipulation Type | -0.08 | 0.45 | 1.78 | 0.25 | 0.50 | 1054 | 0.62 |
| Accuracy Condition x Trust in AI x Manipulation Type x Attitudes | 0.03 | 0.40 | 1.78 | 0.22 | 0.47 | 1054 | 0.64 |

As indicated in the main text, we also conducted the same analyses as above but political ideology as a predictor in the model instead of stance, as shown in Table S9.

**Table S9**

*Accuracy Condition, Trust in AI, Manipulation Type, and Political Ideology Predicting Bias Perception*

| **Predictor Variable** | **B** | **Mean Square** | **full model error term** | **F** | **t** | **df full model** | **p** |
| --- | --- | --- | --- | --- | --- | --- | --- |
| Accuracy Condition | -0.29 | 22.94 | 1.75 | 13.11 | 3.62 | 1055 | <.001 |
| Trust in AI | -0.26 | 126.38 | 1.75 | 72.21 | 8.50 | 1055 | <.001 |
| Manipulation Type | -0.42 | 32.09 | 1.75 | 18.34 | 4.28 | 1055 | <.001 |
| Political Ideology | 0.01 | 0.69 | 1.75 | 0.40 | 0.63 | 1055 | 0.53 |
| Trust in AI x Political Ideology | 0.07 | 36.12 | 1.75 | 20.64 | 4.54 | 1055 | <.001 |
| Accuracy Condition x Political Ideology | 0.04 | 1.54 | 1.75 | 0.88 | 0.94 | 1055 | 0.35 |
| Manipulation Type x Political Ideology | -0.05 | 1.91 | 1.75 | 1.09 | 1.04 | 1055 | 0.30 |
| Accuracy Condition x Trust in AI | -0.22 | 21.07 | 1.75 | 12.04 | 3.47 | 1055 | <.001 |
| Trust in AI x Manipulation Type | -0.26 | 21.47 | 1.75 | 12.27 | 3.50 | 1055 | <.001 |
| Accuracy Condition x Manipulation Type | 0.02 | 0.01 | 1.75 | 0.01 | 0.09 | 1055 | 0.93 |
| Accuracy Condition x Trust in AI x Political Ideology | -0.04 | 2.49 | 1.75 | 1.42 | 1.19 | 1055 | 0.23 |
| Trust in AI x Manipulation Type x Political Ideology | -0.10 | 14.17 | 1.75 | 8.10 | 2.85 | 1055 | 0.00 |
| Accuracy Condition x Manipulation Type x Political Ideology | -0.03 | 0.14 | 1.75 | 0.08 | 0.29 | 1055 | 0.77 |
| Accuracy Condition x Trust in AI x Manipulation Type | -0.09 | 0.61 | 1.75 | 0.35 | 0.59 | 1055 | 0.55 |
| Accuracy Condition x Trust in AI x Manipulation Type x Political Ideology | 0.03 | 0.02 | 1.75 | 0.01 | 0.10 | 1055 | 0.92 |

**Post-correction Bias Perceptions**

***Post-correction Bias Perceptions without Manipulation Type as a Factor***

Participants’ responses to the eight scaled measures of perceived bias were highly correlated (α = .94) and hence were combined to form a single composite (*M* = 3.47, S*D* = 1.41). We conducted a regression analysis wherein stance, accuracy condition, trust in AI, and their interactions served as centered predictors of post-correction perceptions of bias. The results revealed a significant main effect of the accuracy manipulation such that the more accurate system was viewed as less biased, *b* = -.31, *t (*1062) = -3.72, *p* < .001, *r* = .11. There was also a significant main effect of trust in AI on system bias perceptions such that those who were less trusting of AI found the system to be more biased, *b* = -.26, *t*(1062) = -8.29, *p* < .001, *r* = .25. These main effects were qualified by a significant two-way interaction between accuracy condition and trust in AI, *b* = -.22, *t*(1062) = -3.53, *p* < .001, *r* = .11, such that the effect of the accuracy manipulation on perceived bias was stronger with higher trust in AI. Additionally, there was a significant two-way interaction between stance and trust in AI, *b* = -.043, *t*(1062) = -2.52, *p* = .012, *r* = .08 such that the effect of stance on perceived bias was stronger with higher trust in AI. However, no other predictors in the model were significant (*ps* ≥ .285).

***Post-correction Bias Perceptions with Gun Control Attitudes Rather than Stance***

Participants’ responses to the eight scaled measures of perceived bias were highly correlated (α = .94) and hence were combined to form a single composite (*M* = 3.47, S*D* = 1.41). We conducted a regression analysis wherein gun control attitudes, accuracy condition, trust in AI, and their interactions served as centered predictors of post-correction perceptions of bias. The results revealed a significant main effect of the accuracy manipulation such that the more accurate system was viewed as less biased, *b* = -.31, *t (*1062) = -3.63, *p* < .001, *r* = .11. There was also a significant main effect of trust in AI on system bias perceptions such that those who were less trusting of AI found the system to be more biased, *b* = -.27, *t*(1062) = -8.46, *p* < .001, *r* = .26. Additionally, there was a main effect of gun control attitudes, *b* = .07, *t*(1062) = 3.38, *p* < .001, *r* = .10. These main effects were qualified by a significant two-way interaction between accuracy condition and trust in AI, *b* = -.23, *t*(1062) = -3.61, *p* < .001, *r* = .11, such that the effect of the accuracy manipulation on perceived bias was stronger with higher trust in AI. However, no other predictors in the model were significant (*ps* ≥ .26).

***Post-correction Bias Perceptions with Political Ideology Rather than Stance***

Participants’ responses to the eight scaled measures of perceived bias were highly correlated (α = .94) and hence were combined to form a single composite (*M* = 3.47, S*D* = 1.41). We conducted a regression analysis wherein political ideology, accuracy condition, trust in AI, and their interactions served as centered predictors of post-correction perceptions of bias. The results revealed a significant main effect of the accuracy manipulation such that the more accurate system was viewed as less biased, *b* = -.29, *t (*1063) = -3.48, *p* < .001, *r* = .11. There was also a significant main effect of trust in AI on system bias perceptions such that those who were less trusting of AI found the system to be more biased, *b* = -.26, *t*(1063) = -8.53, *p* < .001, *r* = .26. These main effects were qualified by a significant two-way interaction between accuracy condition and trust in AI, *b* = -.21, *t*(1063) = -3.46, *p* < .001, *r* = .11, such that the effect of the accuracy manipulation on perceived bias was stronger with higher trust in AI. Additionally, there was a significant two-way interaction between political ideology and trust in AI, *b* = .080, *t*(1063) = 5.20, *p* < .001, *r* = .16 such that the effect of stance on perceived bias was stronger with higher trust in AI. However, no other predictors in the model were significant (*ps* ≥ .26).
